# Comprehensive Analysis of the Transcriptome-Wide m6A Methylation Modification Difference in Liver Fibrosis Mice by High-Throughput m6A Sequencing

**DOI:** 10.3389/fcell.2021.767051

**Published:** 2021-11-16

**Authors:** Chang Fan, Yanzhen Ma, Sen Chen, Qiumei Zhou, Hui Jiang, Jiafu Zhang, Furong Wu

**Affiliations:** ^1^ Experimental Center of Clinical Research, The First Affiliated Hospital of Anhui University of Chinese Medicine, Hefei, China; ^2^ School of Pharmacy, Anhui University of Chinese Medicine, Hefei, China; ^3^ Key Laboratory of Xin’an Medicine of the Ministry of Education, Anhui University of Chinese Medicine, Hefei, China; ^4^ Department of Pharmacy, The First Affiliated Hospital of Anhui University of Chinese Medicine, Hefei, China; ^5^ Department of Pharmacy, The First Affiliated Hospital of USTC, Division of Life Sciences and Medicine, University of Science and Technology of China, Hefei, China

**Keywords:** m6A methylation, m6A-seq, liver fibrosis, HSCs, WTAP

## Abstract

N6-Methyladenosine (m6A), a unique and common mRNA modification method in eukaryotes, is involved in the occurrence and development of many diseases. Liver fibrosis (LF) is a common response to chronic liver injury and may lead to cirrhosis and even liver cancer. However, the involvement of m6A methylation in the development of LF is still unknown. In this study, we performed a systematic evaluation of hepatic genome-wide m6A modification and mRNA expression by m6A-seq and RNA-seq using LF mice. There were 3,315 genes with significant differential m6A levels, of which 2,498 were hypermethylated and 817 hypomethylated. GO and KEGG analyses illustrated that differentially expressed m6A genes were closely correlated with processes such as the endoplasmic reticulum stress response, PPAR signaling pathway and TGF-β signaling pathway. Moreover, a total of 90 genes had both a significant change in the m6A level and mRNA expression shown by joint analysis of m6A-seq and RNA-seq. Hence, the critical elements of m6A modification, including methyltransferase WTAP, demethylases ALKBH5 and binding proteins YTHDF1 were confirmed by RT-qPCR and Western blot. In an additional cell experiment, we also observed that the decreased expression of WTAP induced the development of LF as a result of promoting hepatic stellate cell (HSC) activation. Therefore, this study revealed unique differential m6A methylation patterns in LF mice and suggested that m6A methylation was associated with the occurrence and course of LF to some extent.

## Introduction

N6-Methyladenosine (M6A) is a posttranscriptional modification found in eukaryotic messenger RNA (mRNA), which is similar to DNA methylation and histone modification and is regulated by a variety of methyltransferases (Bushkin et al., 2019; Gu et al., 2019; Berulava et al., 2020). Methyltransferase complexes are composed of METTL3 (methyltransferase-like 3), METTL14 and their additional linker molecules such as WTAP (Wilms tumor associated protein) and KIAA1429, which can catalyze mRNA m6A methylation. The m6A methylation site on RNA is recognized by m6A-binding proteins, including YTHDC1/2 (1ap2 containing YTH domain), YTHDF1/2/3 (YTH family proteins 1–2–3) and IGF2BP1/2/3 (insulin-like growth factor 2 mRNA binding protein 1/2/3), which can bind to methylated m6A sites and perform specific functions. In addition, demethyltransferase FTO (fat mass and obesity related protein) and ALKBH5 (alkyl B homolog 5) reduce m6A modified RNA to original RNA (Du et al., 2018; Zhang Z. et al., 2020; Mapperley et al., 2021). The combined action of these methyltransferases makes m6A modification a dynamic and reversible process (Lu et al., 2020). It has been confirmed that m6A modification affects the control of key cellular processes, including RNA stability (Wang et al., 2014), translation efficiency (Wang et al., 2015), secondary structure (Liu et al., 2015), subcellular localization (Meyer and Jaffrey, 2014), splicing and transport (Yang et al., 2018), and plays important roles in a variety of diseases (Zhang B. et al., 2020; Liu et al., 2020).

Liver fibrosis (LF) is defined as excessive deposition of extracellular matrix (ECM) in response to various cases of liver injury, which is a reversible abnormal tissue response, and excessive activation of hepatic stellate cells (HSCs) is central to its pathogenesis (Bataller and Brenner, 2005; Zhang et al., 2017; Smith-Cortinez et al., 2020). LF is the most common pathological consequence of liver diseases and may lead to liver cirrhosis and liver cancer, and even develop into liver failure in severe cases (Wang Q. et al., 2020). Existing studies have found that m6A methylation plays an extremely important role in a variety of physiological and pathological processes of the liver (Lin et al., 2020; Ondo et al., 2021). Zhong et al. (2019) found that the m6A binding protein YTHDF2 can inhibit tumor proliferation and growth by reducing the stability of EGFR mRNA in hepatocellular carcinoma. Ma et al. (2017) found that the methyltransferase METTL14 can inhibit the metastasis of hepatocellular carcinoma by regulating the methylation of microRNAs. However, as a preliminary process in these severe liver diseases, m6A methylation in LF is rarely described.

**FIGURE 1:**
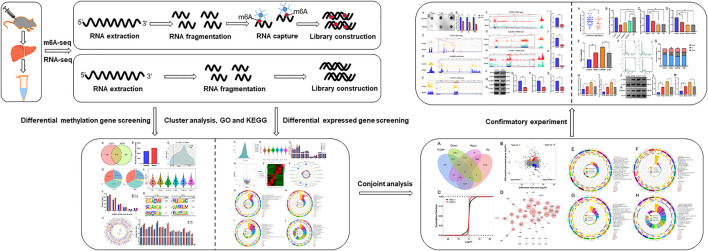
A schematic diagram of m6A-seq and RNA-seq analyses of mice with LF. LF was induced by subcutaneous injection of CCl_4_ in mice, and extracted total RNA from liver. Then, RNA was fragmented, and the m6A RNA was separated by immunoprecipitation magnetic beads specifically recognizing m6A sites. Subsequently, the m6A-seq and RNA-seq library were constructed and sequenced.

The purpose of this study was to establish the expression profile of m6A modification in mice with LF and to explore the potential regulatory mechanism of m6A methylation on LF. Therefore, we used m6A-seq and RNA-seq, to analyze the difference in gene methylation modification and mRNA expression levels after LF at the full transcriptional level, and verified the change in methylase expression and its regulatory role in LF (Figure 1). In conclusion, this study revealed that RNA m6A methylation can play a key role in the pathogenesis of LF by regulating the mRNA expression level of related transcripts. Moreover, methylase affects the occurrence and development of LF by regulating the process of m6A methylation, which could represent an important factor in the process of LF.

## Materials and Methods

### Animal

SPF male C57 BL/6 mice (6–8 weeks old, 20 ± 2 g) were purchased from the Experimental Animal Center of Anhui Province. All mice were raised in the animal facility of the First Affiliated Hospital of Anhui University of Chinese Medicine with an indoor temperature of 18–22°C and humidity of 40–60%, under 12 h alternate dark/light cycles. All mice were allowed food and water freely. Following 1 week of adaptive feeding, a model of LF was established by subcutaneous injection of 0.01 ml/g 20% carbon tetrachloride (CCl_4_) in an olive oil solution in the back flank of the mice twice a week for 12 weeks, as described in our previous study (Fan et al., 2020). The number of samples was three per group for control mice and LF model mice. The experimental design was approved by the Animal Ethics Committee of Anhui University of Chinese Medicine (AHUCM-mouse-2020032).

### Histopathological Analysis

Twelve weeks after establishing the model, the mice were sacrificed by cervical dislocation and the liver samples were taken for histopathological analysis under white light, and hematoxylin and eosin and Masson staining.

Another part of the fresh liver sample was fixed in 2.5% glutaraldehyde and incubated overnight at 4°C. The sample was then fixed in 2% osmium tetroxide for 1 h and dehydrated to 100% through a fractionated series of ethanol (Jiang et al., 2018). Then the sample was embedded in the resin and observed under an electron microscope.

### M6A Sequencing and RNA Sequencing

Total RNA was isolated from mouse liver tissue using TRIzol reagent (Invitrogen, United Statesa) according to the manufacturer’s protocol. In this study, we used an m6A-specific antibody (Sigma-Aldrich, ABE572) for immunoprecipitation RNA. The m6A RNA-seq service was provided by Shanghai Bohao Biotechnology Corporation (Shanghai, China). Briefly, poly (A) RNA was captured by VAHTS 2X Frag/Prime Buffer. Then one part of the RNA fragment was used to construct the RNA-seq library, and the other part was used for m6A RNA immunoprecipitation through the GenSeqTM m6A-MERIP kit (GenSeq Inc., Cyberjaya, Malaysia), which was used to construct the m6A-seq library. All operations were carried out in accordance with the manufacturer’s instructions. RNA input samples without immunoprecipitation and m6A input samples were used for the generation of RNA-seq libraries. The library quality was evaluated with a Bioptic Qsep100 Analyzer (Bioptic lnc., Taiwan, China). Library sequencing was performed on an Illumina NovaSeq instrument with 150 bp paired-end reads.

### Sequencing Data Processing

Cutadapt (v2.5.0) was used to trim adapters and filter for sequences, FastQC (www.bioinformatics.babraham.ac.uk/projects/fastqc) was used to analyze the quality of sequencing data, and the sequencing mass distribution, base content distribution and repeated sequencing fragment proportion were obtained (Garsmeur et al., 2018). Then, the remaining reads were aligned to the human ensemble genome GRCh38 (mouse ensemble genome GRCm38) using Hisat2 aligner (v2.1.0) under the following parameters: -rna-strandness RF. m6A peaks were identified using the exomePeak R package (v2.13.2) under the following parameters: “PEAK_CUTOFF_PVALUE = 0.05, PEAK_CUTOFF_FDR = NA, FRAGMENT_LENGTH = 200”. Identified m6A peaks with a *p* value < 0.05 were chosen for the *de novo* motif analysis using homer (v4.10.4) under the following parameters: “-len 6 -rna”. M6A-RNA-related genomic features were visualized using the Guitar R package (v1.16.0). We used the HOMER (http://homer.ucsd.edu/homer/ngs/peakMotifs.html) software to analyze the motifs of the m6A peaks (Heinz et al., 2010). The screening of differential m6A peaks was also carried out by the exomePeak R package, and the filtering threshold was *p* value <0.05, |fold change| > 2. Moreover, Bam files of sequencing results were visualized using IGV (http://software.broadinstitute.org/software/igv/) (Robinson et al., 2011).

### GO and KEGG Analyses

Differential methylated genes and mRNAs screened according to the above filtering threshold *p* value <0.05, |fold change| > 2 were used for Gene Ontology (GO) and Kyoto Encyclopedia of Genes and Genomes (KEGG) analyses (Ashburner et al., 2000). All analyses were performed using the clusterprofile R package (v3.6.0). Then, the top 20 GO terms and pathways were selected for display according to the *p* value and the degree of enrichment. The figures were generated using OmicShare tools (http://www.omicshare.com/tools).

### Protein-Protein Interaction (PPI) Network Analysis

We conducted a joint analysis of genes with differential expression and differential m6A methylation modification and then used the *p* value and fold change to screen out the genes for PPI analysis. These differentially expressed genes were imported into the STRING database, which contains comprehensive information about interactions between proteins, and was used to determine the interaction relationship between genes (Szklarczyk et al., 2017). The PPI network was constructed based on importing the data into Cytoscape 3.5.1 software, and then, the network was analyzed by Network Analyzer. The genes with interactions with combined scores greater than 0.4 were selected to construct a protein-protein interaction network diagram (Wang X. et al., 2020).

## Validation Experiment

### RNA m6A Dot Blot Analysis

A dot blot assay was performed to compare the difference in total m6A levels in liver samples between the control group and the model group. According to the manufacturer’s instructions, the total RNA, was isolated from the liver sample with TRIzol (Thermo, 15596018) and the RNA sample was placed on the nitrocellulose filter membrane. The membrane was dried and cross-linked with 200,000 μJ/cm^2^ UV twice, washed 3 times with PBST for 5 min each time, and blocked at room temperature for 2 h in 5% skimmed milk. The membrane was transferred to a closed solution containing anti-m6A antibody (ab232905, Abcam) at a dilution of 1: 1,000 and incubated overnight at 4°C. Then, the membrane was rinsed again with PBST for 10 min, sealed in a solution of goat antirabbit IgG combined with HRP (Zs-BIO, ZB-2301) at a dilution of 1: 5,000, incubated at room temperature for 1 h and washed with PBST 3 times. The film was developed with ECL (Western Lightning Plus-ECL, Perkin-Elmer) detection reagent (Thermo, 34094), the signal was detected by chemiluminescence, and the bands were analyzed by ImageJ software.

### Isolation and Culture of Primary Mice HSCs

Mice were anesthetized by intraperitoneal injection of pentobarbital sodium and fixed on the operating table. A middle incision of the lower abdomen was used to open the abdominal cavity and exposed the liver and portal vein. Then, the liver was perfused with preheated HBSS at a uniform speed, the open vein was cut when the liver turned gray, and then 0.05% type IV collagenase perfusion solution was perfused (Nishanth et al., 2013; Kim et al., 2016). After perfusion, the liver was cut out and placed in a Petri dish to clean the liver surface with PBS. Tweezers were used to tear up the liver, and 0.05% type IV collagenase was added to the 37°C incubator to digest the tissue for 30 min, followed by filtering with a 200-mesh strainer. The filtrate was centrifuged at 80, 50 and 40 g gradients, and the cell precipitate was collected. The cells were resuspended in serum containing DMEM and seeded in plates precoated with rat-tail collagen I (Zhang et al., 2012; Vig et al., 2015; Yang et al., 2019). After 4 h, the cell culture medium was replaced with serum-free DMEM to continue culturing, and the results of HSC identification are shown in Supplementary Figure 1.

### Synthesis and Screening of siRNA and Cell Transfection

To suppress the expression of WTAP, the sequence information of WTAP was obtained from the NCBI database, and the specific WTAP small interference RNA (siRNA) sequence was designed and synthesized according to the full-length sequence information. The specific sequence information is shown in Supplementary Table 1. All siRNA sequences were synthesized by Shanghai Jima Biotechnology Co., Ltd. (Shanghai, China). Three dose groups of 50 pmol, 100 and 200 pmol were set for each siRNA to screen the best transfection conditions. The murine HSCs were seeded in 6-well cell culture plates and cultured until the degree of cell fusion reached 60–80% (Wang Z. et al., 2021). Then, WTAP siRNA was transfected into HSCs with Lipofectamine 2000 transfection reagent (Invitrogen). After 24, 48 and 72 h of siRNA transfection, the HSCs were collected and the expression of WTAP was detected by RT-qPCR assay.

### Cell Proliferation Assays and Cell Cycle Analysis

The proliferation of HSCs was detected using a CCK-8 assay. In short, HSCs were trypsinized and resuspended in complete medium, and the cell density was adjusted to 1×10^5^. HSCs were inoculated into 96-well plates at 100 μl per well and cultured for 72 h in a 37°C incubator. Then, 10 μl CCK-8 reagent (BestBio, BB-4202-01) was added to each well, and cells were cultured for another 1 h. The absorbance of each well at 450 nm was measured using a microplate reader. Cell cycle was analyzed by flow cytometry. The HSCs of each group were collected and added to PI staining solution (BestBio, BB-4104) and incubated. The percentage of HSCs in each stage was detected by flow cytometry, and the data were analyzed by FlowJo software (Tree Star Inc., United Statesa).

### RT-qPCR

RT-qPCR was used to detect the expression level of candidate genes. Total RNA from HSCs was extracted with TRIzol (Thermo, 15596018). An ultramicro spectrophotometer was used to determine the concentration and purity of RNA. Then, cDNA reverse transcription and RT-qPCR reactions were performed using the PrimeScript™ RT reagent Kit with gDNA Eraser (TaKaRa, RR047A) and 2×SYBR Green qPCR Master Mix (High ROX) (Servicebio, G3322-05). The primer information is shown in Supplementary Table 2. Reactions proceeded using the following conditions: 95°C for 30 s, followed by 40 cycles of 95°C for 15 s and 60°C for 30 s.

### Western Blot

Total proteins were obtained from HSCs using the radioimmunoprecipitation assay (RIPA) lysis buffer (Beyotime, P0013B) and PMSF (Biosharp, BL507A). The protein contents of the samples were determined by the bicinchoninic acid method. Twenty micrograms of protein samples were separated by 10% SDS-PAGE and transferred to polyvinylidene fluoride membranes. Following blocking with 5% skim milk for 1 h at room temperature, the membranes were incubated with primary antibodies against WTAP (Affinity, DF3282), YTHDF1 (Affinity, DF3422), ALKBH5 (Affinity, DF2585), α-SMA (Affinity, AF1032), and collagen Ⅰ (Affinity, AF7001) overnight at 4°C. The dilution concentrations of the above antibodies were all 1:1,000. After washing with TBST, diluted goat-anti-mouse IgG (1:10,000) antibody (Zs-BIO, ZB-2305) or goat anti-rabbit IgG (1:3,000) antibody (Zs-BIO, ZB-2301) conjugated with horseradish peroxidase was added, and membranes were incubated for 2 h at room temperature. The membranes were developed with an enhanced chemiluminescence detection kit, and the bands were analyzed by ImageJ software.

### Statistical Analysis

The experimental data are presented as the mean ± standard deviation (SD). Statistical analysis was performed by using SPSS 23.0 software. Paired Student’s t-tests were used to detect the differences between the two groups. For multiple comparisons, one-way ANOVA was used with Tukey’s multiple comparisons test. When the *p* value was <0.05, the results were considered to be statistically significant.

## Results

### Pathologic HE Staining, Sirius Red Staining and Transmission Electron Microscopy of the Liver

**FIGURE 2:**
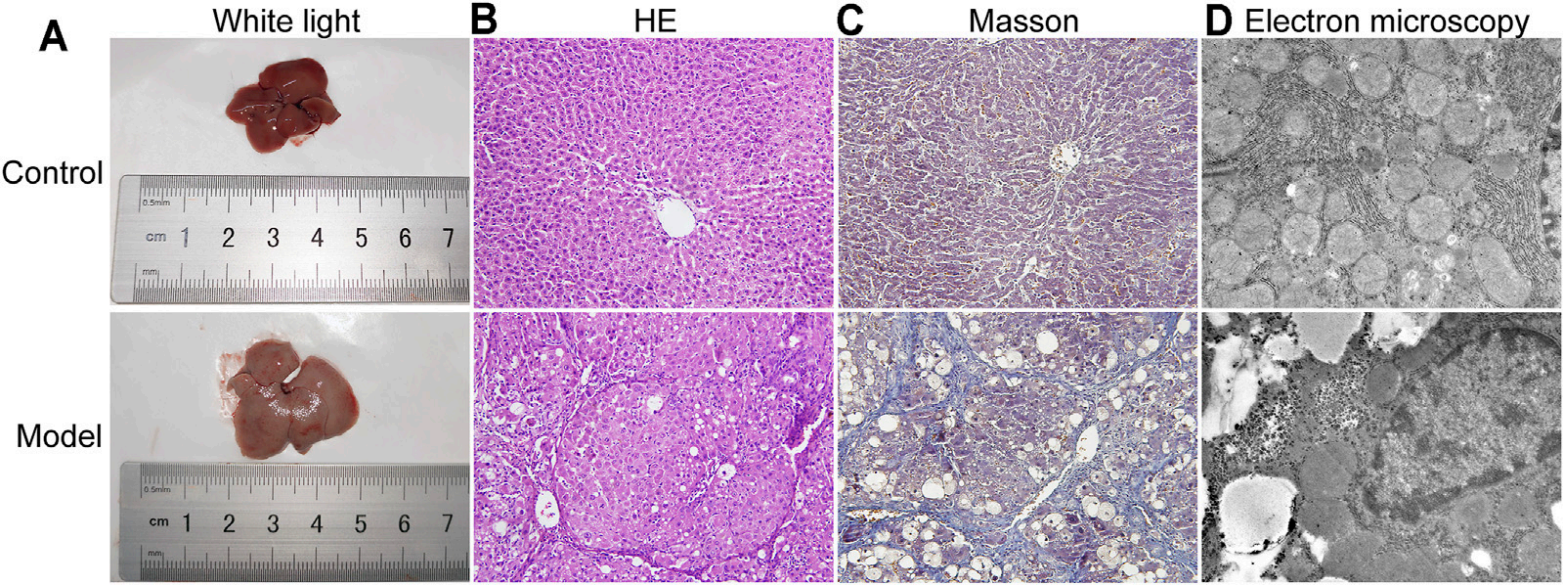
Collected livers were subjected to pathological analysis by white light, HE and Sirius red staining and transmission electron microscopy. **(A)** Liver under white light. **(B)** HE staining (200-fold). **(C)** Sirius Red staining (200-fold). **(D)** Transmission electron microscopy (TEM) analysis (20000-fold).

Liver morphology and the pathological changes in LF mice were observed by white light, HE staining, Masson staining and transmission electron microscopy. As shown in Figure 2A, after 12 weeks of CCl_4_ induction, the livers of the control group were red and smooth, while the livers of the model group were relatively swollen and rough, and the color was gray and white. In Figure 2B, the results of HE staining showed that the structure of the hepatic lobules in the control group was clear, and the hepatocyte cords were in their normal arrangement. In contrast, in the model group there were abundant and large lipid droplets in the cytoplasm of hepatocytes, severe steatosis, disordered liver tissue structure, obvious hyperplasia of fibrotic tissue, and unclear structure of some hepatic lobules.

In Figure 2C, the results of Masson staining showed that there was a large amount of collagen deposition in the liver tissue of the model group compared with the control group. Similarly, obvious changes in the subcellular structure of the liver were observed under an electron microscope (Figure 2D). Hepatocytes in the control group were intact and without morphological signs of degeneration or necrosis, while in the model group, the hepatocytes showed abnormal morphological changes, including disappearance of the cell boundary, rupture of the cell membrane, cytoplasmic turbidity, organelle expansion and nuclear shrinkage.

### General Description of m6A Methylation Modification in LF

**FIGURE 3:**
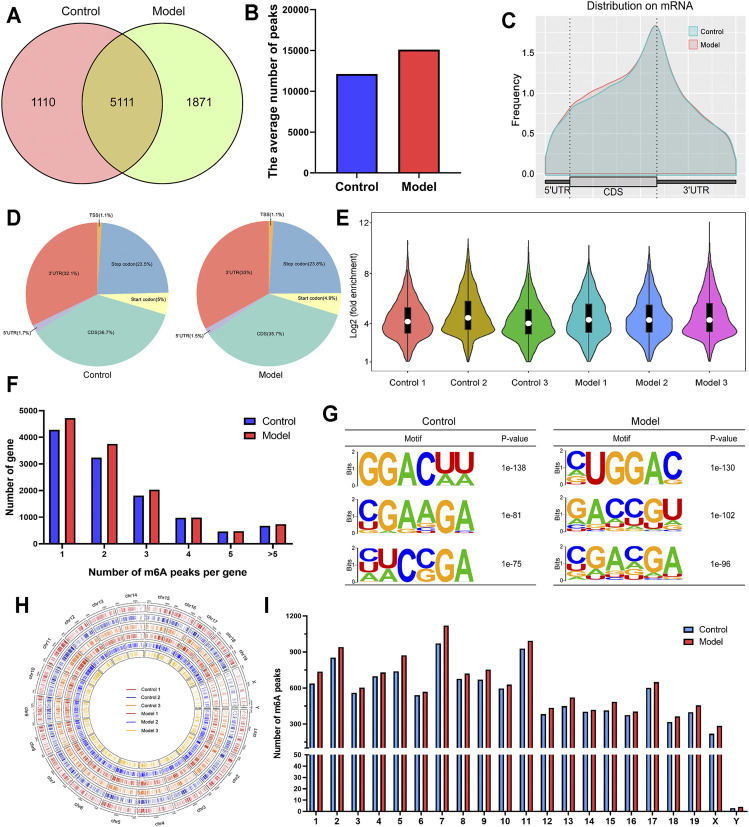
Overview of m6A-modified transcripts in LF mice. **(A)** Venn diagram of m6A-modified genes in the control group and the model group. **(B)** The average number of m6A peaks in each group. **(C)** Density of differential m6A peaks along transcripts. Each transcript was divided into three parts: 5′UTR, CDS, and 3′UTR. **(D)** Pie charts showing the region of m6A peaks in each group. **(E)** Violin plot of the relative abundance of m6A peaks in each group. **(F)** Number of peaks per transcript. **(G)** The most conserved sequence motif of the differential m6A peak region. **(H)** The distribution patterns of m6A peaks in different chromosomes. **(I)** The count of m6A peaks in per chromosome.

We compared m6A methylation peaks at each site in hepatic tissues from mice with fibrosis. The differences and overlaps in m6A methylation between the individuals are shown by the Venn diagram in Figure 3A. We found 6,221 m6A methylation modifier genes in the control group and 6,982 m6A methylation modifier genes in the model group, of which 5,111 m6A methylation modifier genes were common between the two groups. Compared with the control group, 1871 m6A methylation modifier genes appeared, and 1,110 m6A methylation modifier genes disappeared in the model group, indicating that there was a significant difference in the m6A modification pattern after LF. Figure 3B shows the level of m6A methylation in different groups. We found an average of 12166 peaks in the control group and 15100 peaks in the model group.

As shown in Figures 3C,D, m6A methylation of mRNAs occurred mainly in coding sequences (CDSs) and 3′ untranslated regions (3′UTRs). More specifically, approximately 35.7% of m6A peaks were distributed in the CDS region, and 33% of m6A peaks were distributed in the 3′UTR. The violin diagram (Figure 3E) shows the results of the enrichment degree analysis of m6A methylation in each sample. The average logarithmic fold-enrichment of the control group was 4.8, while the average logarithmic fold-enrichment of the model group was 5.3. By means of the distribution of m6A peaks in each gene, we found that approximately 37% of the genes had separate m6A modification sites, and 80% of the genes had one to three m6A modification sites (Figure 3F).

Subsequently, we predicted the m6A motif in LF by the mRNA sequence corresponding to m6A methylation peaks. As shown in Figure 3G, the most significant mRNA methylation occurred at the RRAC motifs. The analysis of the m6A methylation distribution at different chromosome loci found that the m6A peaks of genes in the model group increased, and the chromosomes with the highest m6A methylation frequency were chromosome 7 with 1,119 m6A methylation peaks, chromosome 11 with 993 m6A methylation peaks and chromosome 2 with 940 m6A methylation peaks (Figures 3H,I). By further comparison, we found that there was no significant difference in the distribution number of m6A peaks on chromosomes between the two groups.

### Analysis of Differentially Methylated m6A Genes and Their Signaling Pathways

**FIGURE 4:**
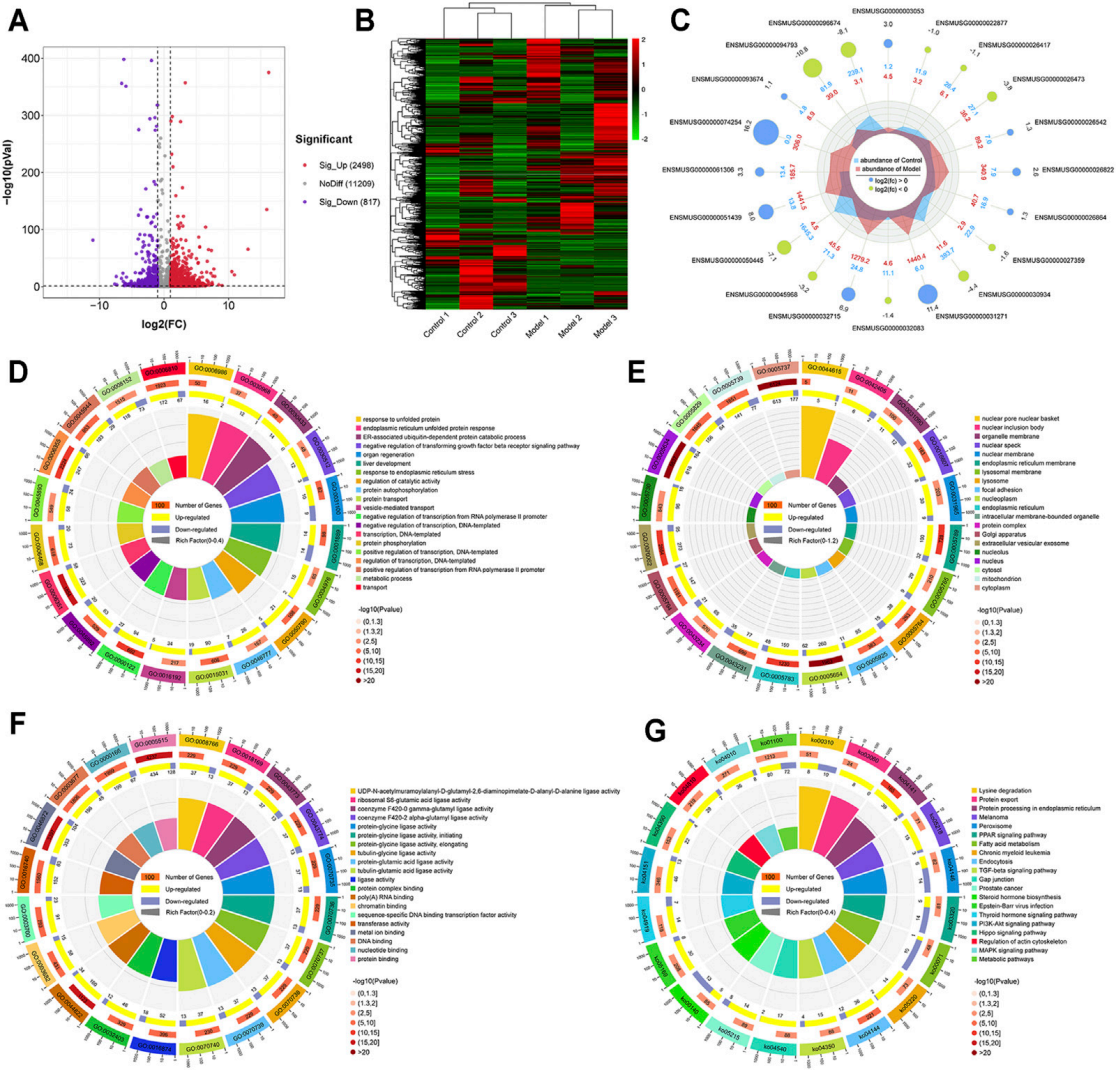
Genes with differential m6A methylation modification in LF. **(A)** Volcano plot representation of microarray data on the differentially expressed m6A methylation genes. The blue and red dots to the left and to the right of the two vertical lines indicate more than a 2-fold change and represent the differentially expressed m6A methylation genes with statistical significance. **(B)** Hierarchical cluster analysis of differentially expressed m6A methylation genes. Hierarchical clustering shows that the differentially expressed m6A methylation genes ultimately cluster into two major branches, including hypermethylated genes, which are labeled in red, and hypomethylated genes, which are labeled in green. The darker the color, the more significant the difference. **(C)** The radar map shows the top 10 most significant hypermethylated genes and top 10 hypomethylated genes. **(D)** GO biological processes enrichment analysis. **(E)** GO cellular component enrichment analysis. **(F)** GO molecular function enrichment analysis. **(G)** KEGG enrichment analysis.

Using the filtering criteria of a *p* value <0.05 and |fold change| >2, 3,315 genes with differential m6A methylation were identified, of which 2,498 m6A hypermethylated genes and 817 m6A hypomethylated genes were identified (Figures 4A,B). We also visually assessed the enrichment degree and fold change of the top 10 hypermethylated genes and top 10 hypomethylated genes (Figure 4C), as shown in Table 1. Specific information of all differentially methylated m6A genes is presented in Supplementary file 1.

**TABLE 1:** the top 10 hypermethylation genes and top 10 hypomethylation genes.

Gene	ID	Description	Chromosome	Start	End	Sizes	*p* Value	Log_2_FC	Class	Hyper/Hypo
Trib3	ENSMUSG00000032715	tribbles pseudokinase 3	2	152337421	152338619	1,198	0	6.93	exon	Hyper
Cd14	ENSMUSG00000051439	CD14 antigen	18	36725103	36726289	1,186	0	8.02	CDS	Hyper
Serpina7	ENSMUSG00000031271	serine (or cysteine) peptidase inhibitor, clade A (alpha-1 antiproteinase, antitrypsin), member 7	X	139080062	139080331	269	0	11.40	3′UTR	Hyper
Cyp2c29	ENSMUSG00000003053	cytochrome P450, family 2, subfamily c, polypeptide 29	19	39330237	39330446	209	0	3.03	3′UTR	Hyper
Hspa5	ENSMUSG00000026864	heat shock protein 5	2	34775567	34776318	751	0	1.30	CDS	Hyper
Cyp2a4	ENSMUSG00000074254	cytochrome P450, family 2, subfamily a, polypeptide 4	7	26314847	26315088	241	0	16.2	3′UTR	Hyper
Lcn2	ENSMUSG00000026822	lipocalin 2	2	32384662	32384871	209	0	2.64	exon	Hyper
Slc38a10	ENSMUSG00000061306	solute carrier family 38, member 10	11	120104735	120106716	1,301,283	0	3.29	CDS	Hyper
Rpl41	ENSMUSG00000093674	ribosomal protein L41	10	128548143	128548497	30,822	0	1.11	exon	Hyper
Apcs	ENSMUSG00000026542	serum amyloid P-component	1	172894048	172895041	662,221	0	1.28	CDS	Hyper
Mup15	ENSMUSG00000096674	major urinary protein 15	4	61435819	61435969	150	0	-8.12	3′UTR	Hypo
Pigr	ENSMUSG00000026417	polymeric immunoglobulin receptor	1	130851592	130852249	657	0	-1.05	3′UTR	Hypo
Teddm2	ENSMUSG00000045968	transmembrane epididymal family member 2	1	153899900	153900228	328	0	-3.22	exon	Hypo
Oat	ENSMUSG00000030934	ornithine aminotransferase	7	132557925	132558254	329	0	-4.41	3′UTR	Hypo
Cyp8b1	ENSMUSG00000050445	cytochrome P450, family 8, subfamily b, polypeptide 1	9	121914355	121916095	1,740	0	-7.06	CDS	Hypo
Hrg	ENSMUSG00000022877	histidine-rich glycoprotein	16	22960759	22961536	777	0	-1.02	CDS	Hypo
Apoa1	ENSMUSG00000032083	apolipoprotein A-I	9	46229224	46230407	45,603	0	-1.38	CDS	Hypo
Glul	ENSMUSG00000026473	glutamate-ammonia ligase (glutamine synthetase)	1	153907866	153908376	510	0	-3.79	CDS	Hypo
Slc27a2	ENSMUSG00000027359	solute carrier family 27 (fatty acid transporter), member 2	2	126587765	126588035	270	0	-1.65	CDS	Hypo
Mup12	ENSMUSG00000094793	major urinary protein 12	4	60737382	60737562	180	0	-10.80	3′UTR	Hypo

Simultaneously, the results of GO and KEGG analyses showed the enrichment of GO functions and pathways of differentially methylated genes. We found 1122 GO terms were significantly enriched in biological processes (Figure 4D), 210 GO terms were significantly enriched in cellular components (Figure 4E), and 476 GO terms were significantly enriched in molecular functions (Figure 4F), especially in the process of transcription, liver development, response of endoplasmic reticulum to unfolded proteins, and protein binding. Similarly, KEGG analysis found that 104 pathways were significantly enriched (Figure 4G), especially protein processing in the endoplasmic reticulum, PI3K-Akt signaling pathway and TGF-β signaling pathway. Specific information on the GO and KEGG pathway enrichment analyses is presented in Supplementary Table 3.

### Description of mRNA Expression and Analysis of Differential Genes in LF

**FIGURE 5:**
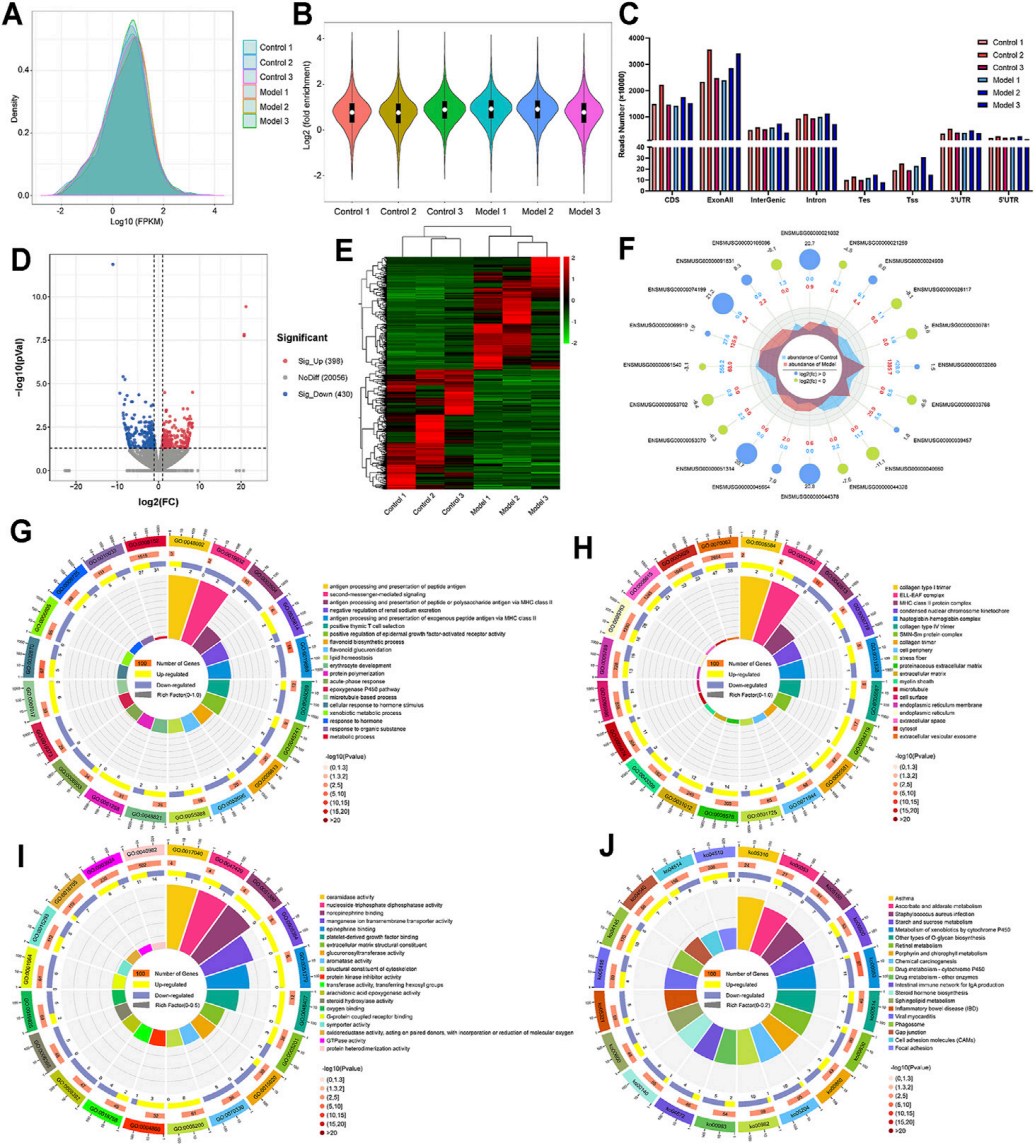
The overall expression of mRNA and the description of differentially expressed mRNAs. **(A)** Metagene plots reveal the distribution intensity and abundance of mRNA expression after sequence alignment. **(B)** Violin plot of the relative abundance of mRNA expression in each sample. **(C)** Regional distribution of mRNA. **(D)** Volcano plot representation of microarray data on the differentially expressed mRNA genes. **(E)** Hierarchical cluster analysis of differentially expressed mRNA genes. **(F)** The radar map shows the top 10 upregulated genes and top 10 downregulated genes. **(G)** GO biological processes enrichment analysis. **(H)** GO cellular component enrichment analysis. **(I)** GO molecular function enrichment analysis. **(J)** KEGG enrichment analysis.

In Figure 5A, not only the mRNA distribution and abundance of control samples and LF samples were shown, but also the peak patterns of these samples were visually displayed. The violin diagram in Figure 5B demonstrates a similar result; the average logarithmic fold-enrichment of the control group was 1.2, while the average logarithmic fold-enrichment of the model group was 1.3. The gene distribution pattern of the control group was also different from the gene distribution pattern of the model group, but they were distributed mainly in the CDS region and exon region (Figure 5C).

Then, similar to the screening of differentially methylated genes, a *p* value <0.05 and |fold change| > 2 were used as screening criteria, and we found 828 differentially expressed genes, including 398 upregulated genes and 430 downregulated genes (Figures 5D,E). Moreover, we also visually compared the expression and corresponding abundance of the top 10 upregulated genes and top 10 downregulated genes (Figure 5F), as shown in Table 2. Specific information of all differentially expressed RNAs is presented in Supplementary File 2. Meanwhile, the results of GO analysis showed that 376 GO terms were significantly enriched in biological processes (Figure 5G), 64 GO terms were significantly enriched in cellular components (Figure 5H), and 136 GO terms were significantly enriched in molecular functions (Figure 5I), particularly in cellular response to hormone stimulus, proteinaceous extracellular matrix, extracellular matrix structural constituent, and more. Similarly, in Figure 4J, the results of KEGG analysis found that 41 pathways were significantly enriched (Figure 4J), particularly the metabolism of xenobiotics by cytochrome P450, retinol metabolism, chemical carcinogenesis, and more. Specific information on the GO and KEGG pathway enrichment analyses is presented in Supplementary Table 4.

**TABLE 2:** the top 10 up-regulated genes and top 10 down-regulated genes.

Gene	ID	Description	Chromosome	Start	End	Sizes	*p* Value	Log_2_FC	Up/Down
Krtdap	ENSMUSG00000074199	keratinocyte differentiation associated protein	7	30487321	30490522	3,201	3.72E-10	21.21	Up
Slc15a5	ENSMUSG00000044378	solute carrier family 15, member 5	6	137960584	138056914	96330	1.45E-08	20.76	Up
Ffar2	ENSMUSG00000051314	free fatty acid receptor 2	7	30517773	30523200	5,427	1.74E-08	20.74	Up
Ngb	ENSMUSG00000021032	neuroglobin	12	87144305	87149313	5,008	1.74E-08	20.74	Up
Gm4707	ENSMUSG00000091831	predicted gene 4,707	17	71765298	71766913	1,615	3.13E-05	8.26	Up
Apoa4	ENSMUSG00000032080	apolipoprotein A-IV	9	4,6151994	46154757	2,763	3.22E-05	1.48	Up
Cdc42ep2	ENSMUSG00000045664	CDC42 effector protein (Rho GTPase binding) 2	19	5965664	5974844	9,180	0.000279,435	7.03	Up
Efemp2	ENSMUSG00000024909	epidermal growth factor-containing fibulin-like extracellular matrix protein 2	19	5523982	5532545	8,563	0.000280,346	5.99	Up
Hba-a1	ENSMUSG00000069919	hemoglobin alpha, adult chain 1	11	32233511	32234465	954	0.000320,964	1.90	Up
Ppl	ENSMUSG00000039457	periplakin	16	4904155	4950285	46130	0.000358,356	1.85	Up
Cyp2b9	ENSMUSG00000040660	cytochrome P450, family 2, subfamily b, polypeptide 9	7	25872836	25910086	37250	1.39416E-12	-11.05	Down
Slc5a2	ENSMUSG00000030781	solute carrier family 5 (sodium/glucose cotransporter), member 2	7	127864829	127871602	6,773	3.95079E-06	-8.58	Down
Gbp10	ENSMUSG00000105096	guanylate-binding protein 10	5	105363565	105387399	23834	5.67824E-06	-8.14	Down
Nebl	ENSMUSG00000053702	nebulette	2	17348720	17736275	387,555	3.48814E-05	-9.35	Down
Cyp46a1	ENSMUSG00000021259	cytochrome P450, family 46, subfamily a, polypeptide 1	12	108300640	108328493	27853	4.29999E-05	-4.78	Down
Trp53i13	ENSMUSG00000044328	transformation related protein 53 inducible protein 13	11	77398925	77406806	7,881	4.80688E-05	-7.60	Down
Zap70	ENSMUSG00000026117	zeta-chain (TCR) associated protein kinase	1	36800879	3,6821899	21020	8.02432E-05	-8.09	Down
Nrxn2	ENSMUSG00000033768	neurexin II	19	6468761	6594199	125,438	0.000108,292	-6.52	Down
Cfap300	ENSMUSG00000053070	cilia and flagella associated protein 300	9	8021673	8042824	21151	0.000109,326	-6.33	Down

### Overview of Transcriptome Profiles and Conjoint Analyses of m6A-Seq and RNA-Seq Data

**FIGURE 6:**
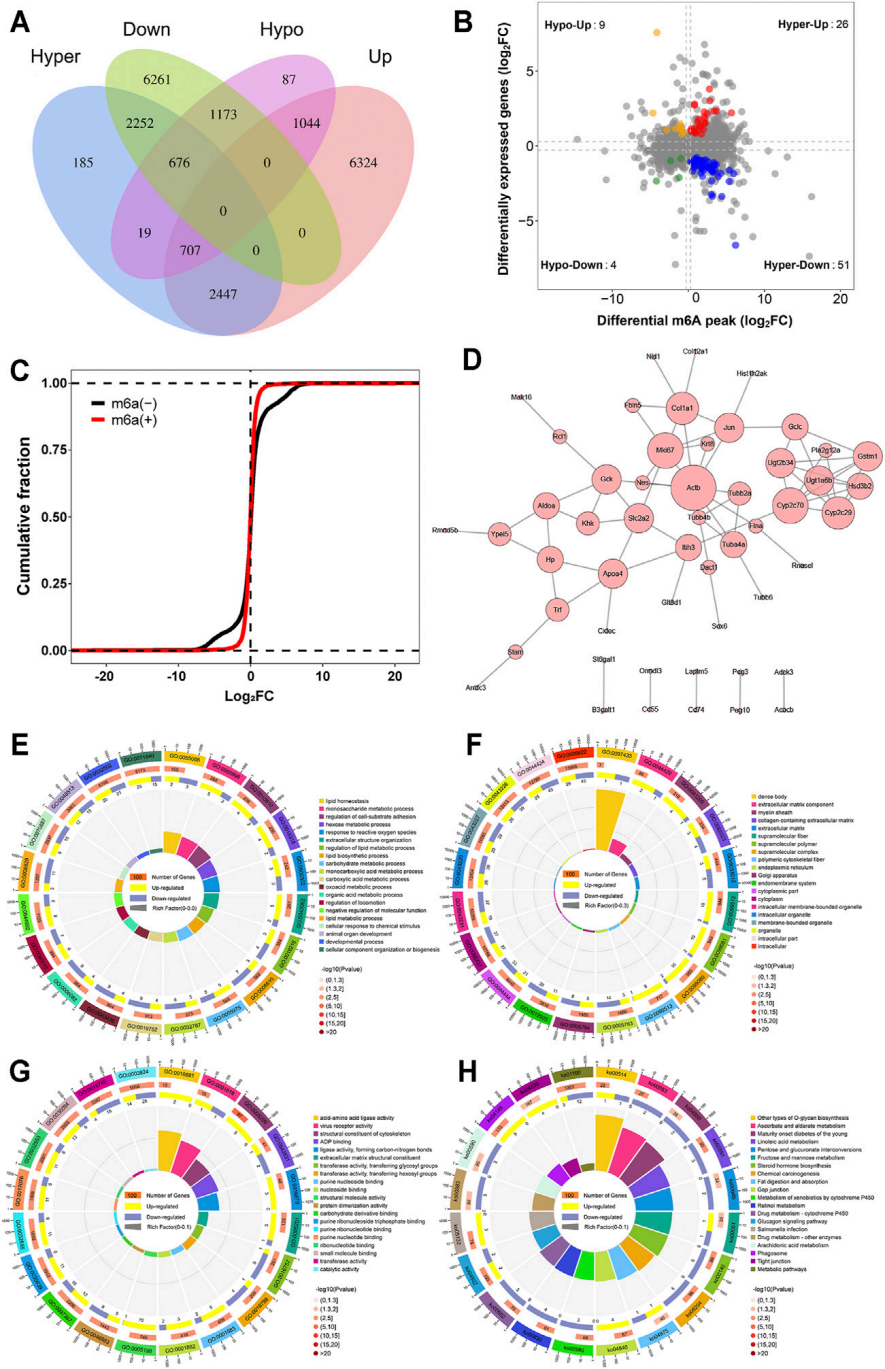
Joint analysis of m6A methylation and mRNA expression. **(A)** Venn diagram of peaks with m6A methylation and mRNA. **(B)** Four quadrant graph of genes with differential m6A methylation and differentially expressed mRNA levels. **(C)** Cumulative frequency plot showing that there was a correlation between differential m6A methylation genes and mRNA levels. **(D)** PPI of genes with differentially expressed m6A methylation and differentially expressed mRNA. **(E)** GO biological processes enrichment analysis. **(F)** GO cellular component enrichment analysis. **(G)** GO molecular function enrichment analysis. **(H)** KEGG enrichment analysis.

A conjoint analysis was conducted for m6A-seq and RNA-seq data. We found that a total of 8,299 peaks located on 2,353 genes not only had m6A modification but also had altered mRNA levels (Figure 6A). However, not all of them were significant. As shown in Figure 6B, by setting the filter conditions of a *p* value < 0.05 and |fold change| >2, we found 90 genes that commonly had significant differential m6A methylation levels and significant differentially expressed mRNA levels. Among these genes, there were 4 genes with m6A hypomethylation and downregulated mRNA expression, 51 genes with m6A hypermethylation and downregulated mRNA expression, 26 genes with m6A hypermethylation and upregulated mRNA expression and 9 genes with m6A hypomethylation and upregulated mRNA expression. The specific information on these genes is shown in Supplementary Table 5.

Subsequently, we confirmed the correlation between m6A modification and mRNA levels. The results in Figure 6C show that differential m6A-methylated transcripts do have different mRNA expression levels; that is, the mRNA expression level of hypomethylated transcripts is often higher than the mRNA expression level of hypermethylated transcripts. Based on interactions with combined scores ≥0.4, the PPI network analysis constructed interaction networks for these differential genes, as shown in Figure 6D.

The results of GO analysis showed that 670 GO terms were significantly enriched in biological processes (Figure 6E), 85 GO terms were significantly enriched in cellular components (Figure 6F), and 148 GO terms were significantly enriched in molecular functions (Figure 6G), particularly in lipid biosynthetic process, endoplasmic reticulum correlation, structural constituent of cytoskeleton, and more. Similarly, in Figure 6H, the results of KEGG analysis found that 29 pathways were significantly enriched, particularly steroid hormone biosynthesis, chemical carcinogenesis, gap junction, and more. The specific information of GO and KEGG pathway enrichment analyses is presented in Supplementary Table 6.

### Levels of m6A Methylation and Methylase Expression in LF

**FIGURE 7:**
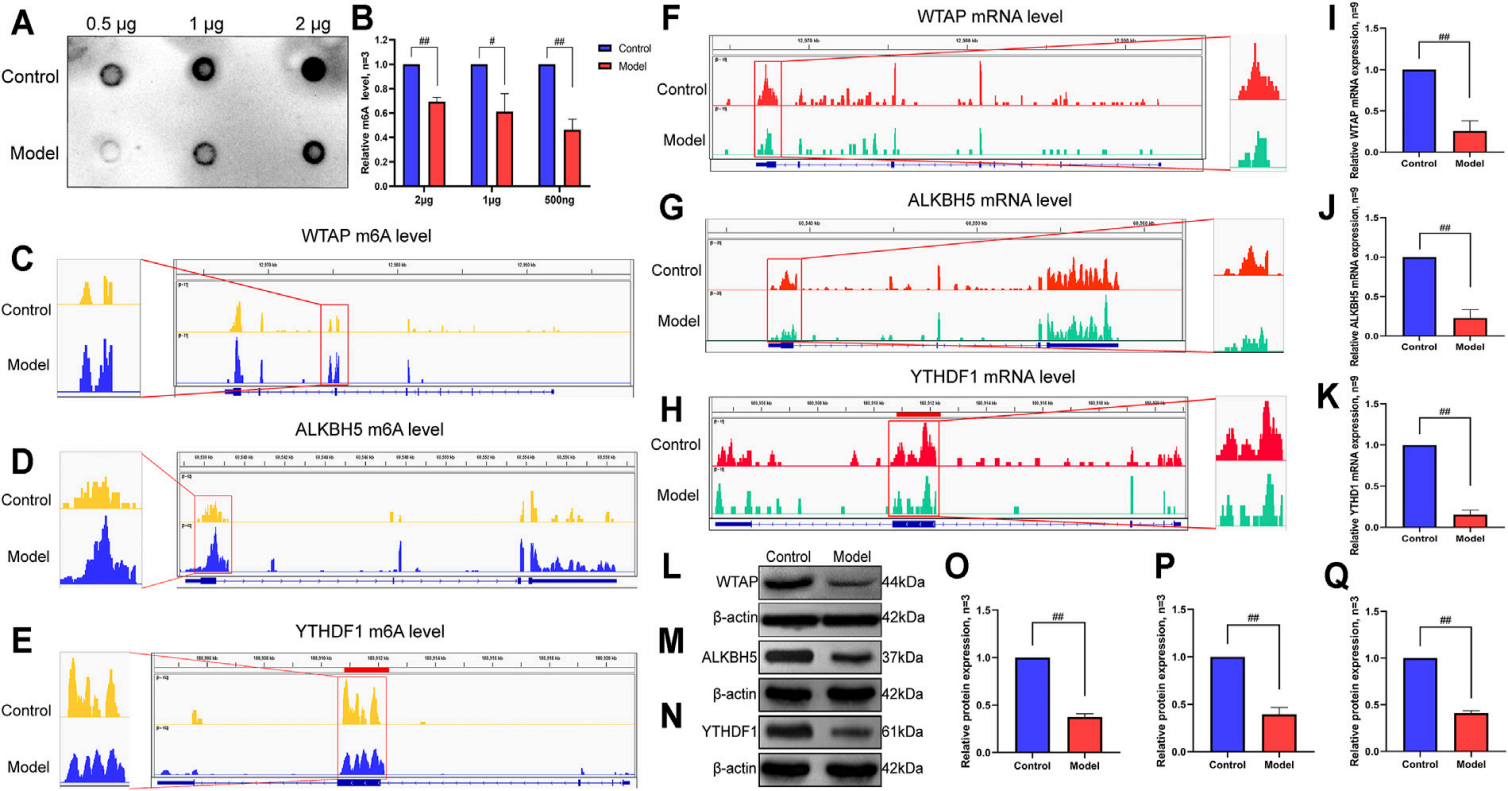
Verification of m6A methylation level and methylase expression in LF. **(A)** The m6A methylation level in LF. **(B)** Semiquantitative analysis of m6A methylation. **(C)** IGV plots of the WTAP m6A level. **(D)** IGV plots of the ALKBH5 m6A level. **(E)** IGV plots of the YTHDF1 m6A level. **(F)** IGV plots of the WTAP expression level. **(G)** IGV plots of the ALKBH5 expression level. **(H)** IGV plots of the YTHDF1 expression level. **(I)** mRNA expression level of WTAP. **(J)** mRNA expression level of ALKBH5. **(K)** mRNA expression level of YTHDF1. **(L)** Protein expression levels of WTAP. **(M)** Protein expression levels of ALKBH5. **(N)** Protein expression levels of YTHDF1. **(O)** Semiquantitative analysis of WTAP protein. **(P)** Semiquantitative analysis of YTHDF1 protein. **(Q)** Semiquantitative analysis of ALKBH5 protein. ^##^ *p* < 0.01 compared with the control group, ^#^ *p* < 0.05 compared with the control group.

To further explore the changes in m6A methylation in LF, we performed an m6A dot blot analysis. The results showed that compared with the control group, the m6A methylation abundance of the model group was significantly decreased (Figures 7A,B). Subsequently, considering that the difference in m6A levels in LF was probably caused by m6A regulatory enzymes, we focused on the methyltransferase WTAP, demethylase ALKBH5 and m6A binding protein YTHDF1. IGV visualization analysis was used to show the sequencing results intuitively. At the m6A methylation level, we found that the m6A levels of WTAP and ALKBH5 increased, while the YTHDF1 level decreased in LF (Figures 7C–E).

Likewise, at the mRNA level, we found that the expression of WTAP, ALKBH5 and YTHDF1 was reduced (Figures 7F–H) by IGV visualization analysis. Then, an RT-qPCR assay was utilized to examine the expression of the above genes. The results showed that the expression levels of WTAP, ALKBH5 and YTHDF1 in the model group were significantly lower than those in the control group, which was consistent with the IGV results (Figures 7I–K). Moreover, we also verified the protein expression levels of WTAP, ALKBH5 and YTHDF1 by Western blot and found that the protein levels of the three genes also decreased significantly in the model group (Figure 7L–Q).

### Effects of Methyltransferase WTAP on Proliferation, Cell Cycle and Activation Markers of HSCs

**FIGURE 8:**
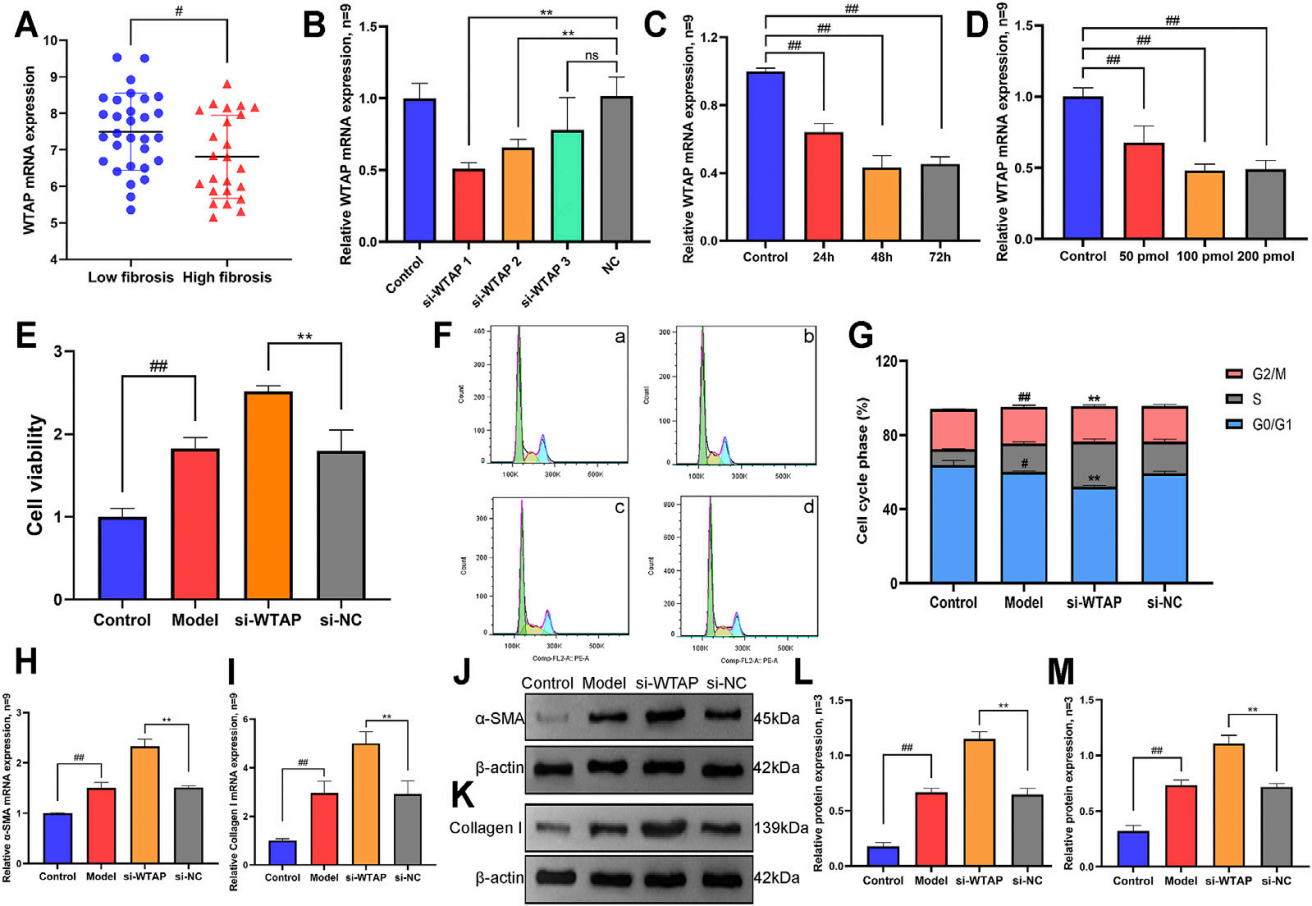
Effects of methyltransferase WTAP on proliferation, cell cycle and activation markers of HSCs. **(A)** Expression levels of WTAP in low-fibrosis and high-fibrosis samples derived from the GEO database. **(B)** Small interfering RNA of WTAP was screened by RT-qPCR assay. **(C)** Optimal stimulation time of WTAP small interfering RNA was screened by RT-qPCR assay. **(D)** Optimal stimulation concentration of WTAP small interference RNA was screened by RT-qPCR assay. **(E)** Cell proliferation was detected by CCK8 assay. **(F)** The phase of the cell cycle was detected by flow cytometry. a, control group. b, model group. c, si-WTAP group. d, si-NC group. **(G)** Quantification of the cell cycle results. **(H)** mRNA expression level of α-SMA. **(I)** The mRNA expression level of collagen Ⅰ. **(J)** Protein expression levels of α-SMA. **(K)** Protein expression levels of collagen Ⅰ. **(L)** Semiquantitative analysis of α-SMA protein. **(M)** Semiquantitative analysis of collagen Ⅰ protein.

As shown in Figure 8A, we analyzed the expression of WTAP in human LF samples through the GEO database (GSE33650) and found that the expression level of WTAP in high-fibrosis samples was significantly lower than the expression level of WTAP in low-fibrosis samples, which was consistent with our present experimental results. Furthermore, we designed and synthesized small interfering RNA targeting WTAP. As shown in Figures 8B–D, we screened the small interfering RNA sequences, durations and concentrations of WTAP small interfering RNA using RT-qPCR and found that the optimal interference sequence was si-WTAP-1, the optimum time of siRNA treatment for interference was 48 h, and the optimum concentration of siRNA for interference was 100 pmol. Follow-up experiments were carried out according to the above conditions.

As shown in Figure 8E, the CCK-8 assay results showed that compared with the control group, the proliferation of HSCs in the model group increased, while the proliferation of HSCs further increased after interfering with the expression of WTAP. Then, flow cytometry was used to detect differences in the HSC cell cycle under WTAP interference (Figures 8F,G). The results showed that the number of HSCs in the G0/G1 phase in the model group was significantly lower than that in the control group, while the number of HSCs in S phase and G2/M phase increased significantly. Compared with the model group, the number of HSCs in G0/G1 phase in the si-WTAP group further decreased, while the number of HSCs in the S phase and G2/M phase further increased. Interfering with WTAP promotes the proliferation of HSCs by inducing S phase and G2/M phase arrest.

Moreover, we also detected the expression of the HSC activation markers α-SMA and collagen Ⅰ. As shown in Figure 8H-8M, the mRNA and protein expression levels of α-SMA and collagen Ⅰ were significantly increased in the model group, while the mRNA and protein expression levels of α-SMA and collagen I were further increased after WTAP interference compared with expression in the model group, which also indicated that WTAP interference significantly promoted the activation of HSCs.

## Discussion

Modifications through m6A methylation modification, as a kind of RNA modification that exists widely in liver disease, has naturally received extensive attention (Wu et al., 2020; Pan et al., 2021). With regard to the effect of m6A methylation on the biological function of liver cells, existing studies have focused on the regulatory mechanism of genes and pathways (Zhang C. et al., 2020; Cao et al., 2021). A study by Zhu Y. et al. (2020) found that METTL3-mediated m6A methylation could be regulated by ASIC1a, which in turn affects the processing of miR-350, thus inducing the activation of HSCs and promoting the occurrence and development of LF. Unlike their studies, our study compared the difference in m6A methylation between the control and LF liver tissue, and confirmed that the m6A modification level changed significantly in LF.

Herein, we first constructed m6A-seq and RNA-seq libraries and investigated the changes in m6A methylation and the expression levels of genes in the liver of mice with hepatic fibrosis by methylated RNA immunoprecipitation combined with next-generation sequencing, and the results were analyzed by bioinformatics. We found 6,221 m6A modification genes in the control group and 6,982 m6A modification genes in the model group. Further analysis identified 3,315 different m6A methylation genes, of which 2,498 m6A hypermethylated genes and 817 m6A hypomethylated genes were identified, suggesting that there are some differences in the occurrence and development of m6A methylation in LF. Interestingly, although the m6A methylation of the gene was different, the distribution of m6A methylation in the control livers was similar to that in the model livers. We found that m6A methylation of most genes was distributed in CDS, 3′UTR and stop codon regions, accounting for 90% of the total. This is consistent with the report of Dominissini et al. (2012), who found that m6A methylation sites are mainly concentrated in long exons, stop codons and 3′UTR regions, and this distribution pattern is highly conserved between humans and mice. This distribution pattern may be related to the function of m6A methylation modification. Dynamic m6A modification in different regions affects biological functions such as splicing, output, stability and translation of mRNA (Wang et al., 2014; Wang and He, 2014; Maity and Das, 2016). Therefore, m6A modification may play an important role in regulating the expression of genes related to hepatic fibrosis.

The m6A methylation site exists mainly in the RRACH motif, which is caused by the binding of m6A methyltransferase with the corresponding consensus sequence (Liu et al., 2018; Zhang Z. et al., 2019). The RNA binding motifs of METTL3, METTL14 and WTAP have been confirmed to be GGAC, GGAC and GACU, which are highly conserved between humans and animals (Liu et al., 2014). When the RRACH sequence is mutated, the single nucleotide polymorphism of the corresponding site changes, which affects m6A methylation. Kane et al. (Kane and Beemon, 1987) found that the mutation from GAC to GAU in the consensus sequence leads to the reversal of m6A methylation in Rous sarcoma virus mRNA transcripts. In the current study, we found many similar m6A consensus motifs in the control and LF tissues, but there were also some differences in the sequences, which further confirmed the emergence of specific m6A methylation sites in the process of LF. However, the RRACH consensus sequence is critical for m6A methylation, but not all RRACH sites in the body will have m6A modification (Gilbert et al., 2016), which corresponds to our results; that is, there are unmutated sequence sites, showing that m6A methylation modification is also regulated by other molecular mechanisms and needs further study.

To better understand the functions of these differentially expressed m6A methylated genes, GO and KEGG distribution analyses were conducted. We found that differential m6A genes were primarily involved in biological processes associated with the endoplasmic reticulum stress response, such as the unfolded protein response and the protein catabolic process, and were also related to the development and regeneration of liver organs. In addition, they were closely related to the PPAR signaling pathway, TGF-β signaling pathway and PI3K-Akt signaling pathway. Endoplasmic reticulum stress refers to the state of protein folding damage caused by the destruction of endoplasmic reticulum homeostasis, and some studies have confirmed that endoplasmic reticulum stress plays a role in the occurrence and development of various liver diseases (Huang et al., 2019; Wu et al., 2021). Virginia et al. (Hernández-Gea et al., 2013) found that oxidative stress disrupts endoplasmic reticulum homeostasis in stellate cells and causes the endoplasmic reticulum to enter a stressed state. To reduce the stress response, hepatic stellate cells initiate an unfolded protein response by limiting the accumulation of unfolded proteins during transient stress, which promotes cell activation and accelerates the development of LF. Peroxisome proliferation-activated receptor (PPAR) belongs to the nuclear hormone receptor family and plays an important role in many biological processes, such as adipogenesis (Lefterova et al., 2014), cell differentiation (Kim et al., 2019), cell growth regulation (Zhang X. et al., 2019) and inflammation (Bougarne et al., 2018). Previous studies have found that the activation of the PPAR pathway can delay the progression of hepatic fibrosis, and its activation can inhibit the transformation of HSCs from a resting state to an activated state (Guo et al., 2005; Anty and Lemoine, 2011). Liu and others have further found that the activation of PPAR-γ can reduce the expression of α-SMA and collagen I in HSCs (Yang et al., 2006). Both the TGF-β and PI3K-Akt signaling pathways are one of the classical signaling pathways involved in the progression of LF. Abnormalities in TGF-β can stimulate HSCs to secrete excessive ECM, and the activity of the PI3K-Akt signaling pathway is significantly correlated with collagen production, HSC proliferation and apoptosis (Shah et al., 2013; Wu et al., 2017). Interestingly, the fibrogenic effects of TGF-β and PI3K-Akt are synergistic to some extent. Runyan et al. (2004) found that TGF-β can not only induce the activation of PI3K/Akt, but also enhance the transcriptional activity of Smad3, the target downstream of TGF-β signaling, thus enhancing the expression of collagen I.

By combining analyses of m6A-seq and RNA-seq data, we discovered 90 genes with differences in their m6A methylation peaks and synchronously differential mRNA expression in LF. The expression of these genes may be regulated by m6A modification of mRNAs. Among the genes with the highest differences, many have been identified to be closely related to the occurrence and development of LF, such as ApoA4 (apolipoprotein A4). Wang Y. et al. (2021) found that ApoA4 may reduce LF and liver injury by inhibiting LF mediators and inflammatory cytokines and suppressing proinflammatory hepatic M1 cell invasion. Although some genes have not been proven to be related to LF, they are involved in fibrosis in other tissues. For example, Ninj1 has been shown to promote the activation of macrophages by enhancing the interaction with epithelial cells, thus enhancing the inflammatory response of macrophages to participate in the occurrence and development of pulmonary fibrosis (Choi et al., 2018). These genes regulated by m6A modification may play key roles in the occurrence and development of LF and may also become an important target for the treatment of LF. However, the specific molecular mechanism of the effect of m6A methylation of these genes on LF is still unclear and needs further exploration and research in the future.

The most prominent finding in our data is that there is a significant difference in m6A modification between the LF and control tissues. The dot blot results also confirmed this significant difference, and we found that the overall level of m6A methylation in LF decreased significantly, which suggested that the modification of the m6A genes affected the progression of LF. A possible explanation for the global change in this m6A modification pattern may be the unique expression of the key m6A regulator or its own methylation modification. Considering that methylases play very important roles in regulating m6A methylation of liver fibrosis, we selected WTAP, ALKBH5 and YTHDF1 as the representative of methyltransferase, demethylase and m6A binding protein for further study, which verify the differences in mRNA and protein expression levels. Interestingly, not only did the expression of WTAP and YTHDF1 decrease in LF, but the expression of the demethylase ALKBH5 also decreased significantly. Combined with the decrease in the overall level of m6A modification in LF, we speculated that the m6A level in the body involves the regulation of a variety of methylases, and the change in one or several methylation enzymes alone cannot be used as a decisive factor in determining the level of m6A methylation. The decrease in the m6A level in LF was because the overall degree of demethylation was greater than the decrease in the m6A level of methylation.

As an important component of the m6A methyltransferase complex, WTAP, unlike METTL3 and METTL14, does not have N6-methyladenine methyltransferase activity but is necessary for effective RNA methylation *in vivo* and for the localization of METTL3 and METTL14 in nuclear spots (Śledź and Jinek, 2016). WTAP has been proven to participate in some basic physiological processes, such as mRNA stability (Horiuchi et al., 2006), organ development (Anderson et al., 2014), cell proliferation, apoptosis and cell cycle regulation (Horiuchi et al., 2013). A recent study by Zhu B. et al. (2020) demonstrated that in a rat model of balloon injury-induced hyperplasia of vascular smooth muscle cells (VSMCs), the expression of WTAP decreased significantly. The suggested mechanism is that WTAP regulates p16INK4a through m6A modification and thus causing abnormal proliferation of VSMCs. Nevertheless, contrary to the above findings that WTAP can inhibit cell proliferation, some other studies have shown different results. A study by Chen et al. (2020) confirmed that WTAP could regulate the stability of HMBOX1 mRNA in an m6A methylation-dependent manner, thereby promoting the proliferation and metastasis of osteosarcoma cells. These studies confirmed that as a pivotal enzyme of m6A modification, WTAP can regulate the m6A methylation level in the body, thus fulfilling functionally different roles in different diseases.

Interestingly, in the present study, we found through sequencing that the m6A level of WTAP was significantly upregulated in LF mice, while the expression of mRNA was reduced. Further verification experiments showed that the mRNA and protein expression levels of WTAP decreased significantly, consistent with the sequencing results. Subsequently, we focused on the effect of WTAP interference on HSCs in LF and found that interfering with WTAP promoted the proliferation of HSCs and increased the expression of α-SMA, a marker of HSC activation and collagen I, the main component of extracellular matrix, which indicated that interfering with WTAP could promote the occurrence and development of LF. Therefore, based on the findings of the above study, we speculated that the possible mechanism of WTAP involved in the development of LF was that WTAP acted as a methyltransferase to affect the m6A level on downstream target genes related to cell proliferation and the cell cycle, thus regulating the mRNA expression levels of these genes and ultimately affecting the occurrence and development of LF. These findings may provide new thoughts and insights for other research on WTAP and m6A methylation in LF.

In summary, our findings established a m6A transcriptome map of LF mice, provided a comprehensive investigation of the potential relationship between m6A methylation and mRNA expression in LF, and revealed the key enzymes of m6A modification, especially WTAP, involved in the occurrence and development of LF.

## Data Availability

The datasets presented in this study can be found in online repositories. The names of the repository/repositories and accession number(s) can be found below: BioProject: PRJNA761579, SRA accession: SRP336482.
