# Supplementary material for: Comprehensive Analysis of the Transcriptome-Wide m6A Methylation Modification Difference in Liver Fibrosis Mice by High-Throughput m6A Sequencing
Source: Front Cell Dev Biol. 2021 Nov 16;9:767051. doi: 10.3389/fcell.2021.767051 (PMC8635166; doi:10.3389/fcell.2021.767051)
Supplement: Supplementary file 1 [file Table1.DOCX]

**Supplementary Table 1** the sequence information of WTAP siRNA

|  | forward sequence | reverse sequence |
| --- | --- | --- |
| siRNA1 | 5’-GGCACGGGAUGAGUUAAUUTT-3’ 5’-AAUUAACUCAUCCCGUGCCTT-3’ | |
| siRNA2 | 5’-UCAUGCGGCUAGCAACCAATT-3’ 5’-UUGGUUGCUAGCCGCAUGATT-3’ | |
| siRNA3 | 5’-GCCCAACUGAGAUCAACAATT-3’ 5’-UUGUUGAUCUCAGUUGGGCTT-3’ | |
