# Supplementary figures and images for: Comprehensive Analysis of the Transcriptome-Wide m6A Methylation Modification Difference in Liver Fibrosis Mice by High-Throughput m6A Sequencing
Source: Front Cell Dev Biol. 2021 Nov 16;9:767051. doi: 10.3389/fcell.2021.767051 (PMC8635166; doi:10.3389/fcell.2021.767051)

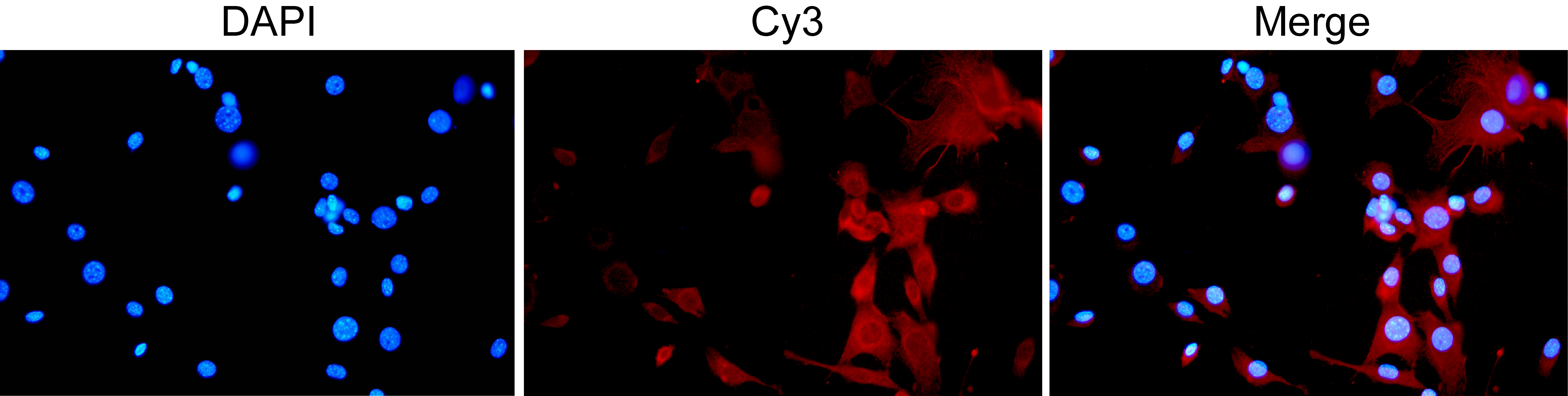

Supplement: Supplementary file 2 [file Image1.TIF]
