# Supplementary material for: Comprehensive Analysis of the Transcriptome-Wide m6A Methylation Modification Difference in Liver Fibrosis Mice by High-Throughput m6A Sequencing
Source: Front Cell Dev Biol. 2021 Nov 16;9:767051. doi: 10.3389/fcell.2021.767051 (PMC8635166; doi:10.3389/fcell.2021.767051)
Supplement: Supplementary file 3 [file Table2.DOCX]

**Supplementary Table 2** Information of primers for RT-qPCR

| Gene | Forward sequence and Reverse sequence | Product length (bp) |
| --- | --- | --- |
| β-actin | F: 5’- AGTGTGACGTTGACATCCGT -3’  R: 5’- TGCTAGGAGCCAGAGCAGTA -3’ | 120 |
| WTAP | F:5’- GCAAGAGTGCACCACTCAAA -3’  R:5’- GCAAGAGTGCACCACTCAAA -3’ | 179 |
| YTHDF1 | F: 5’- CGGCACATCAGACTGGAGAA -3’  R: 5’- TCTTTCCTTACGCACCACCT -3’ | 192 |
| ALKBH5 | F: 5’- GTGACTGTGCTCAGTGGGTA -3’  R: 5’- GGCGATCTGAAGCATAGCTG -3’ | 175 |
