# Supplementary material for: Comprehensive Analysis of the Transcriptome-Wide m6A Methylation Modification Difference in Liver Fibrosis Mice by High-Throughput m6A Sequencing
Source: Front Cell Dev Biol. 2021 Nov 16;9:767051. doi: 10.3389/fcell.2021.767051 (PMC8635166; doi:10.3389/fcell.2021.767051)
Supplement: Supplementary file 4 [file Table5.DOCX]

**Supplementary Table 5** the information of genes with significant differential m6A methylation levels and significant differentially expressed mRNA levels.

| Gene | ID | Gene Description | Log_2_FC-m6A | Log_2_FC-mRNA | P value | Regulation |
| --- | --- | --- | --- | --- | --- | --- |
| Sema6b | ENSMUSG00000001227 | sema domain, transmembrane domain (TM), and cytoplasmic domain, (semaphorin) 6B | 1.24 | 1.59598327 | 0.017686786 | Hyper-Up |
| Tubb6 | ENSMUSG00000001473 | tubulin, beta 6 class V | 2.08 | 2.268853303 | 0.024604178 | Hyper-Up |
| Col1a1 | ENSMUSG00000001506 | collagen, type I, alpha 1 | 3.73 | 2.326648616 | 0.017770937 | Hyper-Up |
| Srpk1 | ENSMUSG00000004865 | serine/arginine-rich protein specific kinase 1 | 0.471 | 0.977086435 | 0.02677773 | Hyper-Up |
| Nes | ENSMUSG00000004891 | nestin | 2.84 | 3.010945837 | 0.019890527 | Hyper-Up |
| Nid1 | ENSMUSG00000005397 | nidogen 1 | 2.49 | 2.172259788 | 0.006775925 | Hyper-Up |
| Pofut2 | ENSMUSG00000020260 | protein O-fucosyltransferase 2 | 0.795 | 1.175266086 | 0.039675283 | Hyper-Up |
| Sik1 | ENSMUSG00000024042 | salt inducible kinase 1 | 2 | 1.107128128 | 0.022505314 | Hyper-Up |
| Stam | ENSMUSG00000026718 | signal transducing adaptor molecule (SH3 domain and ITAM motif) 1 | 0.949 | 0.84979474 | 0.047055974 | Hyper-Up |
| Actb | ENSMUSG00000029580 | actin, beta | 0.404 | 1.156925852 | 0.048813085 | Hyper-Up |
| Cidec | ENSMUSG00000030278 | cell death-inducing DFFA-like effector c | 2.18 | 1.829134987 | 0.041349785 | Hyper-Up |
| Aldoa | ENSMUSG00000030695 | aldolase A, fructose-bisphosphate | 0.884 | 0.897687324 | 0.03509242 | Hyper-Up |
| Flna | ENSMUSG00000031328 | filamin, alpha | 2.2 | 1.537481261 | 0.036751777 | Hyper-Up |
| Mak16 | ENSMUSG00000031578 | MAK16 homolog | 1.53 | 1.324665042 | 0.029084834 | Hyper-Up |
| Ptpn9 | ENSMUSG00000032290 | protein tyrosine phosphatase, non-receptor type 9 | 1.6 | 1.274140111 | 0.029447412 | Hyper-Up |
| Col12a1 | ENSMUSG00000032332 | collagen, type XII, alpha 1 | 5.67 | 2.209227998 | 0.017380338 | Hyper-Up |
| Dennd5a | ENSMUSG00000035901 | DENN/MADD domain containing 5A | 1.74 | 0.790145056 | 0.044386652 | Hyper-Up |
| Tubb4b | ENSMUSG00000036752 | tubulin, beta 4B class IVB | 1.15 | 1.01491987 | 0.032689882 | Hyper-Up |
| Krt8 | ENSMUSG00000049382 | keratin 8 | 2.08 | 1.959311742 | 0.010987688 | Hyper-Up |
| Jun | ENSMUSG00000052684 | jun proto-oncogene | 0.277 | 1.008388847 | 0.038990687 | Hyper-Up |
| Jam2 | ENSMUSG00000053062 | junction adhesion molecule 2 | 2.18 | 1.533564124 | 0.017166638 | Hyper-Up |
| Tubb2a | ENSMUSG00000058672 | tubulin, beta 2A class IIA | 0.818 | 2.794091341 | 0.001480644 | Hyper-Up |
| Phf11d | ENSMUSG00000068245 | PHD finger protein 11D | 2.77 | 3.79336572 | 0.002550908 | Hyper-Up |
| Fastkd5 | ENSMUSG00000079043 | FAST kinase domains 5 | 1.34 | 1.778999494 | 0.033925993 | Hyper-Up |
| Peg10 | ENSMUSG00000092035 | paternally expressed 10 | 3.4 | 2.334869591 | 0.039549332 | Hyper-Up |
| Golt1a | ENSMUSG00000103421 | golgi transport 1A | 0.792 | 2.721808884 | 0.005890567 | Hyper-Up |
| Rmnd5b | ENSMUSG00000001054 | required for meiotic nuclear division 5 homolog B | 3.28 | -1.763178892 | 0.006110319 | Hyper-Down |
| Lgals9 | ENSMUSG00000001123 | lectin, galactose binding, soluble 9 | 1.11 | -0.769433682 | 0.03004448 | Hyper-Down |
| Peg3 | ENSMUSG00000002265 | paternally expressed 3 | 4.26 | -1.830573203 | 0.011414374 | Hyper-Down |
| Cyp2c29 | ENSMUSG00000003053 | cytochrome P450, family 2, subfamily c, polypeptide 29 | 3.03 | -1.615839785 | 0.033036 | Hyper-Down |
| Itih3 | ENSMUSG00000006522 | inter-alpha trypsin inhibitor, heavy chain 3 | 0.917 | -1.048894125 | 0.036137417 | Hyper-Down |
| Abhd1 | ENSMUSG00000006638 | abhydrolase domain containing 1 | 1.82 | -1.272615111 | 0.028822709 | Hyper-Down |
| Hapln4 | ENSMUSG00000007594 | hyaluronan and proteoglycan link protein 4 | 5.4 | -1.617841887 | 0.035046284 | Hyper-Down |
| Slc16a12 | ENSMUSG00000009378 | solute carrier family 16 (monocarboxylic acid transporters), member 12 | 1.68 | -1.802510832 | 0.007126176 | Hyper-Down |
| Pou6f1 | ENSMUSG00000009739 | POU domain, class 6, transcription factor 1 | 3.15 | -1.700186063 | 0.007730008 | Hyper-Down |
| Hyal2 | ENSMUSG00000010047 | hyaluronoglucosaminidase 2 | 1.73 | -1.10039514 | 0.038546261 | Hyper-Down |
| Rab32 | ENSMUSG00000019832 | RAB32, member RAS oncogene family | 1.4 | -1.033424622 | 0.022528304 | Hyper-Down |
| Tcp11l2 | ENSMUSG00000020034 | t-complex 11 (mouse) like 2 | 3.38 | -1.297460555 | 0.01584594 | Hyper-Down |
| Fbln5 | ENSMUSG00000021186 | fibulin 5 | 5.38 | -2.366305821 | 0.036451499 | Hyper-Down |
| Glt8d1 | ENSMUSG00000021916 | glycosyltransferase 8 domain containing 1 | 2.56 | -1.352202438 | 0.023493109 | Hyper-Down |
| Ank | ENSMUSG00000022265 | progressive ankylosis | 2 | -1.62526139 | 0.001518743 | Hyper-Down |
| St6gal1 | ENSMUSG00000022885 | beta galactoside alpha 2,6 sialyltransferase 1 | 1.47 | -0.952073202 | 0.031982142 | Hyper-Down |
| Cd74 | ENSMUSG00000024610 | CD74 antigen (invariant polypeptide of major histocompatibility complex, class II antigen-associated) | 1.74 | -1.052408748 | 0.029968598 | Hyper-Down |
| Mettl7b | ENSMUSG00000025347 | methyltransferase like 7B | 1.07 | -0.939184163 | 0.0396768 | Hyper-Down |
| Coq8a | ENSMUSG00000026489 | coenzyme Q8A | 0.593 | -1.669670798 | 0.024896718 | Hyper-Down |
| Tmem141 | ENSMUSG00000026939 | transmembrane protein 141 | 3.18 | -3.35082049 | 0.001645892 | Hyper-Down |
| Slc2a2 | ENSMUSG00000027690 | solute carrier family 2 (facilitated glucose transporter), member 2 | 0.832 | -1.422272036 | 0.04943239 | Hyper-Down |
| Pla2g12a | ENSMUSG00000027999 | phospholipase A2, group XIIA | 3.36 | -1.269732687 | 0.004448608 | Hyper-Down |
| Laptm5 | ENSMUSG00000028581 | lysosomal-associated protein transmembrane 5 | 1.74 | -1.224239154 | 0.028545156 | Hyper-Down |
| Khk | ENSMUSG00000029162 | ketohexokinase | 0.737 | -1.243314334 | 0.033701102 | Hyper-Down |
| Ugt2b34 | ENSMUSG00000029260 | UDP glucuronosyltransferase 2 family, polypeptide B34 | 2.58 | -1.045758619 | 0.017862107 | Hyper-Down |
| Nup210 | ENSMUSG00000030091 | nucleoporin 210 | 3.35 | -1.120070383 | 0.028970919 | Hyper-Down |
| Prss8 | ENSMUSG00000030800 | protease, serine 8 (prostasin) | 4.4 | -3.364665749 | 0.04065698 | Hyper-Down |
| Mki67 | ENSMUSG00000031004 | antigen identified by monoclonal antibody Ki 67 | 5.95 | -1.846609107 | 0.012514323 | Hyper-Down |
| Hp | ENSMUSG00000031722 | haptoglobin | 0.487 | -1.090759652 | 0.032870272 | Hyper-Down |
| Trf | ENSMUSG00000032554 | transferrin | 0.857 | -0.861123419 | 0.039702697 | Hyper-Down |
| Tut4 | ENSMUSG00000034610 | terminal uridylyl transferase 4 | 2.26 | -0.926864111 | 0.00967948 | Hyper-Down |
| B3galt1 | ENSMUSG00000034780 | UDP-Gal:betaGlcNAc beta 1,3-galactosyltransferase, polypeptide 1 | 3.91 | -2.043317745 | 0.005178226 | Hyper-Down |
| Ninj1 | ENSMUSG00000037966 | ninjurin 1 | 1.76 | -1.345342117 | 0.003444005 | Hyper-Down |
| Ormdl3 | ENSMUSG00000038150 | ORM1-like 3 (S. cerevisiae) | 1.98 | -0.95488629 | 0.029059203 | Hyper-Down |
| Lyplal1 | ENSMUSG00000039246 | lysophospholipase-like 1 | 1.39 | -1.313921291 | 0.025008834 | Hyper-Down |
| Ypel5 | ENSMUSG00000039770 | yippee like 5 | 1.15 | -0.932887246 | 0.017692546 | Hyper-Down |
| Etfrf1 | ENSMUSG00000040370 | electron transfer flavoprotein regulatory factor 1 | 0.789 | -1.317229431 | 0.017198725 | Hyper-Down |
| Gck | ENSMUSG00000041798 | glucokinase | 1.77 | -1.55678457 | 0.015119278 | Hyper-Down |
| Adora1 | ENSMUSG00000042429 | adenosine A1 receptor | 3.51 | -1.602064921 | 0.002488968 | Hyper-Down |
| Dpm3 | ENSMUSG00000042737 | dolichyl-phosphate mannosyltransferase polypeptide 3 | 3.69 | -1.241534936 | 0.036305564 | Hyper-Down |
| Fam25c | ENSMUSG00000043681 | family with sequence similarity 25, member C | 3.01 | -3.233188905 | 0.006102571 | Hyper-Down |
| Dact1 | ENSMUSG00000044548 | dishevelled-binding antagonist of beta-catenin 1 | 3.06 | -2.337737836 | 0.020529305 | Hyper-Down |
| Sox6 | ENSMUSG00000051910 | SRY (sex determining region Y)-box 6 | 2.65 | -1.409543041 | 0.010550412 | Hyper-Down |
| Gstm1 | ENSMUSG00000058135 | glutathione S-transferase, mu 1 | 2.56 | -1.428819549 | 0.020955849 | Hyper-Down |
| Cyp2c70 | ENSMUSG00000060613 | cytochrome P450, family 2, subfamily c, polypeptide 70 | 2.86 | -1.507651367 | 0.004198566 | Hyper-Down |
| H2ac15 | ENSMUSG00000063021 | H2A clustered histone 15 | 6.21 | -6.617903171 | 0.002963746 | Hyper-Down |
| Rnasel | ENSMUSG00000066800 | ribonuclease L (2', 5'-oligoisoadenylate synthetase-dependent) | 3.04 | -1.621175267 | 0.041672274 | Hyper-Down |
| Pex11g | ENSMUSG00000069633 | peroxisomal biogenesis factor 11 gamma | 1.65 | -1.005575951 | 0.047934629 | Hyper-Down |
| Arrdc3 | ENSMUSG00000074794 | arrestin domain containing 3 | 1.87 | -2.035114841 | 0.001763625 | Hyper-Down |
| Esrp2 | ENSMUSG00000084128 | epithelial splicing regulatory protein 2 | 0.316 | -1.061719287 | 0.023005527 | Hyper-Down |
| Ugt1a6b | ENSMUSG00000090145 | UDP glucuronosyltransferase 1 family, polypeptide A6B | 1.8 | -1.438530545 | 0.014232792 | Hyper-Down |
| Dusp3 | ENSMUSG00000003518 | dual specificity phosphatase 3 (vaccinia virus phosphatase VH1-related) | -0.882 | 0.779586388 | 0.042461273 | Hypo-Up |
| Ttll8 | ENSMUSG00000022388 | tubulin tyrosine ligase-like family, member 8 | -4.14 | 7.569052472 | 0.001665912 | Hypo-Up |
| Ehd1 | ENSMUSG00000024772 | EH-domain containing 1 | -0.521 | 0.92300898 | 0.020293231 | Hypo-Up |
| Tuba4a | ENSMUSG00000026202 | tubulin, alpha 4A | -1.01 | 1.162096972 | 0.004949597 | Hypo-Up |
| Cd55 | ENSMUSG00000026399 | CD55 molecule, decay accelerating factor for complement | -1.68 | 1.147582494 | 0.010332791 | Hypo-Up |
| Apoa4 | ENSMUSG00000032080 | apolipoprotein A-IV | -1.06 | 1.48379029 | 3.22142E-05 | Hypo-Up |
| Gclc | ENSMUSG00000032350 | glutamate-cysteine ligase, catalytic subunit | -1.08 | 1.172898946 | 0.030432863 | Hypo-Up |
| Acacb | ENSMUSG00000042010 | acetyl-Coenzyme A carboxylase beta | -2.84 | 1.057472762 | 0.01296246 | Hypo-Up |
| H2-T10 | ENSMUSG00000079491 | histocompatibility 2, T region locus 10 | -4.66 | 2.195854832 | 0.007830584 | Hypo-Up |
| Fgfr4 | ENSMUSG00000005320 | fibroblast growth factor receptor 4 | -0.988 | -0.841709711 | 0.038101389 | Hypo-Down |
| Enho | ENSMUSG00000028445 | energy homeostasis associated | -1.21 | -2.111067651 | 0.020255628 | Hypo-Down |
| Necab1 | ENSMUSG00000040536 | N-terminal EF-hand calcium binding protein 1 | -2.33 | -0.988454947 | 0.038337928 | Hypo-Down |
| Hsd3b2 | ENSMUSG00000063730 | hydroxy-delta-5-steroid dehydrogenase, 3 beta- and steroid delta-isomerase 2 | -4.19 | -2.340754733 | 0.012919453 | Hypo-Down |
