# Supplementary material for: Comprehensive Analysis of the Transcriptome-Wide m6A Methylation Modification Difference in Liver Fibrosis Mice by High-Throughput m6A Sequencing
Source: Front Cell Dev Biol. 2021 Nov 16;9:767051. doi: 10.3389/fcell.2021.767051 (PMC8635166; doi:10.3389/fcell.2021.767051)
Supplement: Supplementary file 5 [file Table3.DOCX]

**Supplementary Table 3-1** GO biological processes enrichment.

| ID | classification | All gene | P value | Differential gene |
| --- | --- | --- | --- | --- |
| GO:0006351 | transcription, DNA-templated | 1854 | 4.73E-16 | 274 |
| GO:0006355 | regulation of transcription, DNA-templated | 2224 | 1.86E-13 | 305 |
| GO:0000122 | negative regulation of transcription from RNA polymerase II promoter | 692 | 1.69E-11 | 119 |
| GO:0015031 | protein transport | 606 | 3.71E-10 | 104 |
| GO:0001889 | liver development | 98 | 1.51E-07 | 27 |
| GO:0006986 | response to unfolded protein | 50 | 2.23E-07 | 18 |
| GO:0045892 | negative regulation of transcription, DNA-templated | 529 | 2.12E-06 | 82 |
| GO:0050790 | regulation of catalytic activity | 108 | 4.15E-06 | 26 |
| GO:0006468 | protein phosphorylation | 619 | 5.69E-06 | 91 |
| GO:0006810 | transport | 1923 | 6.18E-06 | 233 |
| GO:0030433 | ER-associated ubiquitin-dependent protein catabolic process | 40 | 7.23E-06 | 14 |
| GO:0031100 | organ regeneration | 62 | 7.79E-06 | 18 |
| GO:0045944 | positive regulation of transcription from RNA polymerase II promoter | 953 | 9.08E-06 | 128 |
| GO:0030968 | endoplasmic reticulum unfolded protein response | 37 | 1.46E-05 | 13 |
| GO:0008152 | metabolic process | 1515 | 1.88E-05 | 187 |
| GO:0016192 | vesicle-mediated transport | 217 | 4.05E-05 | 39 |
| GO:0046777 | protein autophosphorylation | 167 | 5.44E-05 | 32 |
| GO:0034976 | response to endoplasmic reticulum stress | 65 | 6.09E-05 | 17 |
| GO:0030512 | negative regulation of transforming growth factor beta receptor signaling pathway | 48 | 7.42E-05 | 14 |
| GO:0045893 | positive regulation of transcription, DNA-templated | 549 | 8.35E-05 | 78 |

**Supplementary Table 3-2** GO cellular component enrichment.

| ID | classification | All gene | P value | Differential gene |
| --- | --- | --- | --- | --- |
| GO:0005634 | nucleus | 5837 | 8.11131E-29 | 761 |
| GO:0005737 | cytoplasm | 6124 | 2.27193E-26 | 780 |
| GO:0005654 | nucleoplasm | 1963 | 1.43391E-22 | 312 |
| GO:0070062 | extracellular vesicular exosome | 2664 | 3.24663E-15 | 364 |
| GO:0005783 | endoplasmic reticulum | 1230 | 2.29262E-14 | 196 |
| GO:0005789 | endoplasmic reticulum membrane | 728 | 2.32598E-13 | 130 |
| GO:0005794 | Golgi apparatus | 1161 | 7.48256E-10 | 171 |
| GO:0043231 | intracellular membrane-bounded organelle | 699 | 8.02215E-09 | 112 |
| GO:0005829 | cytosol | 1645 | 8.61351E-08 | 216 |
| GO:0005739 | mitochondrion | 1651 | 1.75566E-07 | 215 |
| GO:0016607 | nuclear speck | 183 | 2.64296E-07 | 40 |
| GO:0005925 | focal adhesion | 383 | 7.35873E-07 | 66 |
| GO:0044615 | nuclear pore nuclear basket | 5 | 7.03095E-06 | 5 |
| GO:0005764 | lysosome | 293 | 9.67456E-06 | 51 |
| GO:0042405 | nuclear inclusion body | 11 | 1.43166E-05 | 7 |
| GO:0043234 | protein complex | 570 | 1.68385E-05 | 84 |
| GO:0031965 | nuclear membrane | 203 | 2.46894E-05 | 38 |
| GO:0031090 | organelle membrane | 100 | 3.74458E-05 | 23 |
| GO:0005765 | lysosomal membrane | 210 | 0.000118078 | 37 |
| GO:0005730 | nucleolus | 843 | 0.000119769 | 111 |

**Supplementary Table 3-3** GO molecular function enrichment.

| ID | classification | All gene | P value | Differential gene |
| --- | --- | --- | --- | --- |
| GO:0005515 | protein binding | 4237 | 1.97726E-18 | 549 |
| GO:0044822 | poly(A) RNA binding | 1123 | 1.76163E-16 | 190 |
| GO:0046872 | metal ion binding | 3397 | 8.49164E-16 | 447 |
| GO:0000166 | nucleotide binding | 1999 | 1.53166E-08 | 259 |
| GO:0016740 | transferase activity | 1560 | 3.52312E-08 | 209 |
| GO:0070739 | protein-glutamic acid ligase activity | 229 | 7.46154E-08 | 48 |
| GO:0070737 | protein-glycine ligase activity, elongating | 229 | 7.46154E-08 | 48 |
| GO:0070738 | tubulin-glycine ligase activity | 229 | 7.46154E-08 | 48 |
| GO:0070735 | protein-glycine ligase activity | 229 | 7.46154E-08 | 48 |
| GO:0070736 | protein-glycine ligase activity, initiating | 229 | 7.46154E-08 | 48 |
| GO:0018169 | ribosomal S6-glutamic acid ligase activity | 229 | 7.46154E-08 | 48 |
| GO:0043774 | coenzyme F420-2 alpha-glutamyl ligase activity | 229 | 7.46154E-08 | 48 |
| GO:0043773 | coenzyme F420-0 gamma-glutamyl ligase activity | 229 | 7.46154E-08 | 48 |
| GO:0008766 | UDP-N-acetylmuramoylalanyl-D-glutamyl-2,6-diaminopimelate-D-alanyl-D-alanine ligase activity | 229 | 7.46154E-08 | 48 |
| GO:0070740 | tubulin-glutamic acid ligase activity | 230 | 8.58749E-08 | 48 |
| GO:0003677 | DNA binding | 1806 | 2.8858E-07 | 231 |
| GO:0016874 | ligase activity | 396 | 6.19568E-07 | 68 |
| GO:0003682 | chromatin binding | 431 | 3.37639E-06 | 70 |
| GO:0003700 | sequence-specific DNA binding transcription factor activity | 793 | 3.52072E-06 | 113 |
| GO:0032403 | protein complex binding | 329 | 3.69101E-06 | 57 |

**Supplementary Table 3-4** KEGG enrichment.

| ID | classification | All gene | P value | Differential gene |
| --- | --- | --- | --- | --- |
| ko04141 | Protein processing in endoplasmic reticulum | 165 | 2.98837E-11 | 47 |
| ko04144 | Endocytosis | 221 | 7.21053E-07 | 47 |
| ko00310 | Lysine degradation | 51 | 2.92098E-05 | 16 |
| ko04151 | PI3K-Akt signaling pathway | 346 | 4.92312E-05 | 59 |
| ko04146 | Peroxisome | 82 | 0.000170083 | 20 |
| ko05218 | Melanoma | 71 | 0.00021164 | 18 |
| ko05169 | Epstein-Barr virus infection | 208 | 0.000268863 | 38 |
| ko03320 | PPAR signaling pathway | 81 | 0.00041989 | 19 |
| ko04350 | TGF-beta signaling pathway | 88 | 0.001240411 | 19 |
| ko04540 | Gap junction | 88 | 0.001240411 | 19 |
| ko04010 | MAPK signaling pathway | 271 | 0.001276392 | 44 |
| ko05215 | Prostate cancer | 89 | 0.001429491 | 19 |
| ko03060 | Protein export | 24 | 0.001944481 | 8 |
| ko00140 | Steroid hormone biosynthesis | 85 | 0.002077678 | 18 |
| ko05220 | Chronic myeloid leukemia | 73 | 0.002493293 | 16 |
| ko01100 | Metabolic pathways | 1213 | 0.004105415 | 151 |
| ko04810 | Regulation of actin cytoskeleton | 219 | 0.004997965 | 35 |
| ko04390 | Hippo signaling pathway | 153 | 0.00654447 | 26 |
| ko00071 | Fatty acid metabolism | 48 | 0.008002782 | 11 |
| ko04919 | Thyroid hormone signaling pathway | 118 | 0.008166434 | 21 |
