# Supplementary material for: Comprehensive Analysis of the Transcriptome-Wide m6A Methylation Modification Difference in Liver Fibrosis Mice by High-Throughput m6A Sequencing
Source: Front Cell Dev Biol. 2021 Nov 16;9:767051. doi: 10.3389/fcell.2021.767051 (PMC8635166; doi:10.3389/fcell.2021.767051)
Supplement: Supplementary file 6 [file Table4.DOCX]

**Supplementary Table 4-1** GO biological processes enrichment.

| ID | classification | All gene | P value | Differential gene |
| --- | --- | --- | --- | --- |
| GO:0032870 | cellular response to hormone stimulus | 67 | 2.56369E-06 | 10 |
| GO:0019886 | antigen processing and presentation of exogenous peptide antigen via MHC class II | 14 | 9.94705E-06 | 5 |
| GO:0048002 | antigen processing and presentation of peptide antigen | 3 | 1.16033E-05 | 3 |
| GO:0002504 | antigen processing and presentation of peptide or polysaccharide antigen via MHC class II | 10 | 4.92788E-05 | 4 |
| GO:0051258 | protein polymerization | 31 | 6.00734E-05 | 6 |
| GO:0008152 | metabolic process | 1515 | 6.34985E-05 | 58 |
| GO:0052696 | flavonoid glucuronidation | 20 | 6.88245E-05 | 5 |
| GO:0009813 | flavonoid biosynthetic process | 20 | 6.88245E-05 | 5 |
| GO:0006953 | acute-phase response | 34 | 0.000103589 | 6 |
| GO:0006805 | xenobiotic metabolic process | 65 | 0.000107522 | 8 |
| GO:0045059 | positive thymic T cell selection | 12 | 0.000112032 | 4 |
| GO:0048821 | erythrocyte development | 24 | 0.000175064 | 5 |
| GO:0010033 | response to organic substance | 111 | 0.000219374 | 10 |
| GO:0007017 | microtubule-based process | 39 | 0.000228334 | 6 |
| GO:0019373 | epoxygenase P450 pathway | 29 | 0.000445509 | 5 |
| GO:0019932 | second-messenger-mediated signaling | 2 | 0.000513567 | 2 |
| GO:0035814 | negative regulation of renal sodium excretion | 8 | 0.000596785 | 3 |
| GO:0055088 | lipid homeostasis | 19 | 0.000773306 | 4 |
| GO:0009725 | response to hormone | 68 | 0.000869791 | 7 |
| GO:0045741 | positive regulation of epidermal growth factor-activated receptor activity | 9 | 0.000880124 | 3 |

**Supplementary Table 4-2** GO cellular component enrichment.

| ID | classification | All gene | P value | Differential gene |
| --- | --- | --- | --- | --- |
| GO:0042613 | MHC class II protein complex | 11 | 2.57424E-06 | 5 |
| GO:0005578 | proteinaceous extracellular matrix | 300 | 2.24715E-05 | 20 |
| GO:0031012 | extracellular matrix | 240 | 0.000145883 | 16 |
| GO:0005581 | collagen trimer | 87 | 0.000172707 | 9 |
| GO:0034719 | SMN-Sm protein complex | 17 | 0.000514758 | 4 |
| GO:0032783 | ELL-EAF complex | 2 | 0.000525926 | 2 |
| GO:0005584 | collagen type I trimer | 2 | 0.000525926 | 2 |
| GO:0070062 | extracellular vesicular exosome | 2664 | 0.000978592 | 85 |
| GO:0071944 | cell periphery | 58 | 0.002092663 | 6 |
| GO:0005783 | endoplasmic reticulum | 1230 | 0.002430006 | 44 |
| GO:0005789 | endoplasmic reticulum membrane | 728 | 0.003028537 | 29 |
| GO:0005829 | cytosol | 1645 | 0.003170848 | 55 |
| GO:0043209 | myelin sheath | 182 | 0.003316859 | 11 |
| GO:0001725 | stress fiber | 65 | 0.003734109 | 6 |
| GO:0005874 | microtubule | 304 | 0.004702082 | 15 |
| GO:0000778 | condensed nuclear chromosome kinetochore | 5 | 0.005022851 | 2 |
| GO:0031838 | haptoglobin-hemoglobin complex | 5 | 0.005022851 | 2 |
| GO:0005615 | extracellular space | 1245 | 0.005063122 | 43 |
| GO:0009986 | cell surface | 558 | 0.005266885 | 23 |
| GO:0005587 | collagen type IV trimer | 6 | 0.00742009 | 2 |

**Supplementary Table 4-3** GO molecular function enrichment.

| ID | classification | All gene | P value | Differential gene |
| --- | --- | --- | --- | --- |
| GO:0005200 | structural constituent of cytoskeleton | 61 | 6.60513E-05 | 8 |
| GO:0005201 | extracellular matrix structural constituent | 36 | 0.000141336 | 6 |
| GO:0046982 | protein heterodimerization activity | 502 | 0.000210632 | 25 |
| GO:0015020 | glucuronosyltransferase activity | 30 | 0.000515523 | 5 |
| GO:0016758 | transferase activity, transferring hexosyl groups | 49 | 0.000792213 | 6 |
| GO:0019825 | oxygen binding | 69 | 0.000928009 | 7 |
| GO:0015293 | symporter activity | 112 | 0.001002176 | 9 |
| GO:0070330 | aromatase activity | 36 | 0.001220243 | 5 |
| GO:0048407 | platelet-derived growth factor binding | 12 | 0.002167012 | 3 |
| GO:0003924 | GTPase activity | 230 | 0.002315074 | 13 |
| GO:0001664 | G-protein coupled receptor binding | 61 | 0.002506306 | 6 |
| GO:0017040 | ceramidase activity | 4 | 0.002966807 | 2 |
| GO:0051380 | norepinephrine binding | 4 | 0.002966807 | 2 |
| GO:0047429 | nucleoside-triphosphate diphosphatase activity | 4 | 0.002966807 | 2 |
| GO:0008392 | arachidonic acid epoxygenase activity | 47 | 0.004053346 | 5 |
| GO:0008395 | steroid hydroxylase activity | 48 | 0.004442278 | 5 |
| GO:0005384 | manganese ion transmembrane transporter activity | 5 | 0.004870769 | 2 |
| GO:0004860 | protein kinase inhibitor activity | 32 | 0.005610168 | 4 |
| GO:0016705 | oxidoreductase activity, acting on paired donors, with incorporation or reduction of molecular oxygen | 119 | 0.005662065 | 8 |
| GO:0051379 | epinephrine binding | 6 | 0.007197153 | 2 |

**Supplementary Table 4-4** KEGG enrichment.

| ID | classification | All gene | P value | Differential gene |
| --- | --- | --- | --- | --- |
| ko00980 | Metabolism of xenobiotics by cytochrome P450 | 97 | 7.40044E-06 | 12 |
| ko00830 | Retinol metabolism | 90 | 2.01516E-05 | 11 |
| ko05204 | Chemical carcinogenesis | 95 | 3.37469E-05 | 11 |
| ko04145 | Phagosome | 170 | 3.5069E-05 | 15 |
| ko00982 | Drug metabolism - cytochrome P450 | 99 | 4.97325E-05 | 11 |
| ko05150 | Staphylococcus aureus infection | 51 | 0.000327125 | 7 |
| ko00140 | Steroid hormone biosynthesis | 85 | 0.000350858 | 9 |
| ko00500 | Starch and sucrose metabolism | 53 | 0.000416747 | 7 |
| ko05416 | Viral myocarditis | 89 | 0.000495104 | 9 |
| ko04510 | Focal adhesion | 208 | 0.001060695 | 14 |
| ko00983 | Drug metabolism - other enzymes | 64 | 0.001318547 | 7 |
| ko00514 | Other types of O-glycan biosynthesis | 49 | 0.001620204 | 6 |
| ko04514 | Cell adhesion molecules (CAMs) | 158 | 0.002779227 | 11 |
| ko05310 | Asthma | 24 | 0.003191253 | 4 |
| ko05321 | Inflammatory bowel disease (IBD) | 59 | 0.004208215 | 6 |
| ko00053 | Ascorbate and aldarate metabolism | 27 | 0.004958474 | 4 |
| ko00860 | Porphyrin and chlorophyll metabolism | 43 | 0.004981981 | 5 |
| ko04672 | Intestinal immune network for IgA production | 46 | 0.006661423 | 5 |
| ko04540 | Gap junction | 88 | 0.007973627 | 7 |
| ko00600 | Sphingolipid metabolism | 48 | 0.007979677 | 5 |
