# Supplementary material for: Comprehensive Analysis of the Transcriptome-Wide m6A Methylation Modification Difference in Liver Fibrosis Mice by High-Throughput m6A Sequencing
Source: Front Cell Dev Biol. 2021 Nov 16;9:767051. doi: 10.3389/fcell.2021.767051 (PMC8635166; doi:10.3389/fcell.2021.767051)
Supplement: Supplementary file 7 [file Table6.DOCX]

**Supplementary Table 6-1** GO biological processes enrichment.

| ID | classification | All gene | P value | Differential gene |
| --- | --- | --- | --- | --- |
| GO:0005996 | monosaccharide metabolic process | 268 | 1.20E-05 | 8 |
| GO:0019752 | carboxylic acid metabolic process | 913 | 6.56E-05 | 13 |
| GO:0008610 | lipid biosynthetic process | 562 | 8.16E-05 | 10 |
| GO:0005975 | carbohydrate metabolic process | 568 | 8.90E-05 | 10 |
| GO:0043436 | oxoacid metabolic process | 954 | 0.000102084 | 13 |
| GO:0044092 | negative regulation of molecular function | 1120 | 0.000134193 | 14 |
| GO:0006082 | organic acid metabolic process | 984 | 0.000138945 | 13 |
| GO:0040012 | regulation of locomotion | 994 | 0.000153556 | 13 |
| GO:0032502 | developmental process | 6199 | 0.000156928 | 41 |
| GO:0010810 | regulation of cell-substrate adhesion | 206 | 0.000178558 | 6 |
| GO:0043062 | extracellular structure organization | 297 | 0.000188848 | 7 |
| GO:0048513 | animal organ development | 3463 | 0.000309031 | 27 |
| GO:0071840 | cellular component organization or biogenesis | 6173 | 0.000327385 | 40 |
| GO:0055088 | lipid homeostasis | 150 | 0.00034147 | 5 |
| GO:0019318 | hexose metabolic process | 236 | 0.000370526 | 6 |
| GO:0070887 | cellular response to chemical stimulus | 2937 | 0.000379032 | 24 |
| GO:0000302 | response to reactive oxygen species | 242 | 0.000423328 | 6 |
| GO:0032787 | monocarboxylic acid metabolic process | 573 | 0.000471761 | 9 |
| GO:0006629 | lipid metabolic process | 1267 | 0.000474214 | 14 |
| GO:0019216 | regulation of lipid metabolic process | 354 | 0.000543986 | 7 |

**Supplementary Table 6-2** GO cellular component enrichment.

| ID | classification | All gene | P value | Differential gene |
| --- | --- | --- | --- | --- |
| GO:0005737 | cytoplasm | 10758 | 3.00E-05 | 64 |
| GO:0044444 | cytoplasmic part | 8445 | 4.62E-05 | 54 |
| GO:0005783 | endoplasmic reticulum | 1686 | 5.55E-05 | 19 |
| GO:0012505 | endomembrane system | 3816 | 9.90E-05 | 31 |
| GO:0097433 | dense body | 7 | 0.000356898 | 2 |
| GO:0062023 | collagen-containing extracellular matrix | 334 | 0.000498942 | 7 |
| GO:0031012 | extracellular matrix | 444 | 0.000537486 | 8 |
| GO:0099512 | supramolecular fiber | 944 | 0.000543347 | 12 |
| GO:0099081 | supramolecular polymer | 949 | 0.000569368 | 12 |
| GO:0099080 | supramolecular complex | 950 | 0.000574697 | 12 |
| GO:0044424 | intracellular part | 13789 | 0.000611243 | 72 |
| GO:0044420 | extracellular matrix component | 46 | 0.000937248 | 3 |
| GO:0043229 | intracellular organelle | 12104 | 0.001098668 | 65 |
| GO:0005622 | intracellular | 13998 | 0.001154049 | 72 |
| GO:0043209 | myelin sheath | 210 | 0.001876731 | 5 |
| GO:0043226 | organelle | 12413 | 0.002628025 | 65 |
| GO:0099513 | polymeric cytoskeletal fiber | 717 | 0.003025986 | 9 |
| GO:0005595 | collagen type XII trimer | 1 | 0.004174092 | 1 |
| GO:0030934 | anchoring collagen complex | 1 | 0.004174092 | 1 |
| GO:0033565 | ESCRT-0 complex | 1 | 0.004174092 | 1 |

**Supplementary Table 6-3** GO molecular function enrichment.

| ID | classification | All gene | P value | Differential gene |
| --- | --- | --- | --- | --- |
| GO:0005200 | structural constituent of cytoskeleton | 67 | 7.68E-06 | 5 |
| GO:0097367 | carbohydrate derivative binding | 2290 | 8.64E-05 | 22 |
| GO:0016757 | "transferase activity, transferring glycosyl groups" | 281 | 0.00014538 | 7 |
| GO:0005198 | structural molecule activity | 746 | 0.000208085 | 11 |
| GO:0003824 | catalytic activity | 5956 | 0.000452287 | 39 |
| GO:0043531 | ADP binding | 41 | 0.00061196 | 3 |
| GO:0036094 | small molecule binding | 2655 | 0.000711543 | 22 |
| GO:0016879 | "ligase activity, forming carbon-nitrogen bonds" | 46 | 0.00085882 | 3 |
| GO:0001883 | purine nucleoside binding | 408 | 0.001341302 | 7 |
| GO:0035639 | purine ribonucleoside triphosphate binding | 1910 | 0.001518171 | 17 |
| GO:0001882 | nucleoside binding | 418 | 0.001540348 | 7 |
| GO:0016758 | "transferase activity, transferring hexosyl groups" | 209 | 0.001608376 | 5 |
| GO:0005201 | extracellular matrix structural constituent | 128 | 0.001827303 | 4 |
| GO:0016881 | acid-amino acid ligase activity | 16 | 0.001875465 | 2 |
| GO:0046983 | protein dimerization activity | 1443 | 0.001891392 | 14 |
| GO:0032555 | purine ribonucleotide binding | 1985 | 0.002293635 | 17 |
| GO:0016740 | transferase activity | 2347 | 0.002338635 | 19 |
| GO:0017076 | purine nucleotide binding | 1998 | 0.002457331 | 17 |
| GO:0032553 | ribonucleotide binding | 2001 | 0.002496475 | 17 |
| GO:0001618 | virus receptor activity | 19 | 0.002651539 | 2 |

**Supplementary Table 6-4** KEGG enrichment.

| ID | classification | All gene | P value | Differential gene |
| --- | --- | --- | --- | --- |
| ko00140 | Steroid hormone biosynthesis | 88 | 0.00012633 | 5 |
| ko05204 | Chemical carcinogenesis | 96 | 0.000190603 | 5 |
| ko04540 | Gap junction | 87 | 0.001402119 | 4 |
| ko00830 | Retinol metabolism | 91 | 0.001656142 | 4 |
| ko04922 | Glucagon signaling pathway | 103 | 0.002608141 | 4 |
| ko00591 | Linoleic acid metabolism | 50 | 0.002736065 | 3 |
| ko04145 | Phagosome | 173 | 0.002751698 | 5 |
| ko01100 | Metabolic pathways | 1353 | 0.005678516 | 15 |
| ko00980 | Metabolism of xenobiotics by cytochrome P450 | 68 | 0.00651868 | 3 |
| ko00982 | Drug metabolism - cytochrome P450 | 70 | 0.007064555 | 3 |
| ko00514 | Other types of O-glycan biosynthesis | 23 | 0.007305431 | 2 |
| ko05132 | Salmonella infection | 78 | 0.009515421 | 3 |
| ko00053 | Ascorbate and aldarate metabolism | 27 | 0.009991296 | 2 |
| ko04950 | Maturity onset diabetes of the young | 28 | 0.01072149 | 2 |
| ko00983 | Drug metabolism - other enzymes | 89 | 0.01360681 | 3 |
| ko00590 | Arachidonic acid metabolism | 90 | 0.01402119 | 3 |
| ko04530 | Tight junction | 167 | 0.01419666 | 4 |
| ko00040 | Pentose and glucuronate interconversions | 34 | 0.01557512 | 2 |
| ko00051 | Fructose and mannose metabolism | 35 | 0.01646033 | 2 |
| ko04975 | Fat digestion and absorption | 40 | 0.02119773 | 2 |
